# VlPAG/DRN Microglia Drive Neuropathic Pain‐Induced Depression via a Defined Neuroimmune Axis

**DOI:** 10.1002/advs.77071

**Published:** 2026-08-11

**Authors:** Jing Yang, Bing‐Jie Tang, Jia‐Yi Li, Shu‐Lan Xie, Shumin Duan, Junping Chen, Shengmei Zhu, Zhen‐Zhong Xu

**Affiliations:** ^1^ Department of Anesthesiology of First Affiliated Hospital and Liangzhu Laboratory School of Brain Science and Brain Medicine Zhejiang University School of Medicine Hangzhou China; ^2^ NANHU Brain‐Computer Interface Institute Hangzhou China; ^3^ School of Public Health Hangzhou Medical College Hangzhou Zhejiang China; ^4^ Department of Anesthesiology Ningbo No. 2 Hospital Wenzhou Medical University Ningbo China; ^5^ MOE Frontier Science Center for Brain Science and Brain‐machine Integration State Key Laboratory of Brain‐machine Intelligence Zhejiang University Hangzhou China; ^6^ NHC and CAMS Key Laboratory of Medical Neurobiology Zhejiang Key Laboratory of Pain Perception and Neuromodulation Zhejiang University Hangzhou China

**Keywords:** biology, chemistry, medicine

## Abstract

Neuropathic pain is frequently comorbid with anxiety and depression, yet the mechanisms linking immune signaling to affective brain circuits remain poorly understood. Here, we identify a neuroimmune circuit in which peripheral nerve injury activates microglia in the midbrain ventrolateral periaqueductal gray/dorsal raphe (vlPAG/DRN), triggering an NLRP3–IL‐1β‐dependent inflammatory cascade. Direct optogenetic or chemogenetic activation of vlPAG/DRN microglia is sufficient to drive negative affective behaviors. We show that local VGLUT2^+^ glutamatergic neurons (vlPAG/DRN^Glu^) are the principal IL‐1R1–expressing targets; IL‐1β activates these neurons to drive anxiety‐ and depression‐like states. Conversely, microglia‐specific *Nlrp3* deletion or local IL‐1R1 blockade prevents neuropathic pain–induced affective deficits. Furthermore, circuit mapping and functional manipulation reveal an excitatory vlPAG/DRN^Glu^ to the bed nucleus of the stria terminalis (BNST^GABA^) pathway that is both sufficient to induce and required to maintain the affective component of neuropathic pain. Together, our findings delineate a microglia–vlPAG/DRN^Glu^–BNST^GABA^ axis that translates peripheral injury into maladaptive emotional states, revealing a discrete neuroimmune circuit substrate for mood comorbidity in chronic pain.

## Graphical abstract

Peripheral nerve injury (SNI) induces neuropathic pain, activates midbrain microglia, and triggers an NLRP3–IL‐1β–dependent inflammatory cascade. IL‐1β signals through IL‐1R1 on vlPAG/DRN VGLUT2^+^ neurons (vlPAG/DRN^Glu^), inducing hyperexcitability and driving the vlPAG/DRN^Glu^→BNST^GABA^ circuit to produce the affective component of neuropathic pain, including depression‐like behaviors.

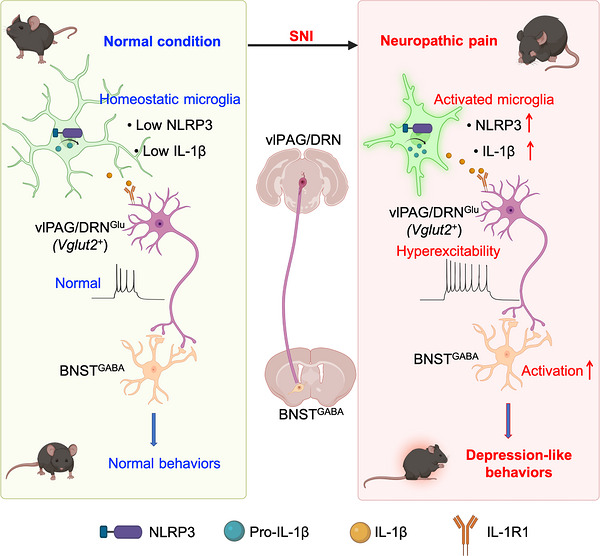

## 1 Introduction

Chronic pain often evolves from a protective signal into a debilitating condition characterized by comorbid anxiety and depression [1, 2, 3]. Although rodent models indicate that persistent pain remodels affective brain circuits [4, 5], the specific neuroimmune mechanisms that convert nociceptive input into sustained negative affect remain a critical gap in knowledge.

Microglia, the brain's resident immune cells, regulate mood and are activated in neuropsychiatric disorders [6, 7, 8, 9, 10]. Notably, the NLRP3 inflammasome—a key microglial sensor that drives IL‐1β maturation and release—is linked to depression, and its inhibition improves affective behavior [11, 12, 13, 14]. However, whether and where NLRP3–IL‐1β signaling directly contributes to pain‐related affective dysfunction is unknown.

The ventrolateral periaqueductal gray and dorsal raphe nucleus (vlPAG/DRN) is a conserved midbrain hub for pain and affect [15, 16, 17, 18]. Its dense connections to threat‐processing regions position it to transform persistent nociception into negative emotionality. While a dorsomedial prefrontal cortex (dmPFC) projection to the vlPAG exerts anxiolytic control [19], activating specific vlPAG/DRN glutamatergic neurons promotes anxiety, suggesting functionally distinct neuronal subpopulations with opposing affective roles. Downstream, the bed nucleus of the stria terminalis (BNST) is critical for sustained anxiety and is dysregulated in chronic pain [4, 20, 21]. However, the source of heightened inhibitory tone in the BNST during pain states, and whether it is driven by a specific vlPAG/DRN projection, remains undefined. We therefore hypothesized that neuropathic pain engages a microglial NLRP3–IL‐1β axis within the vlPAG/DRN to activate a dedicated glutamatergic subpopulation, which in turn drives affective pathology via a selective projection to the BNST.

Here, we identify this precise neuroimmune circuit in which microglial NLRP3–IL‐1β signaling in the vlPAG/DRN drives neuropathic pain‐induced negative affect via local VGLUT2^+^ neurons. These neurons project to BNST GABAergic cells, forming an excitatory vlPAG/DRN VGLUT2^+^ → BNST GABA pathway that is both sufficient to elicit and required to sustain affective comorbidities. Through integrated optogenetic, chemogenetic, conditional knockout, and circuit‐mapping approaches, we delineate a complete microglia–vlPAG/DRN–BNST axis, revealing a discrete neuroimmune substrate for mood dysfunction in chronic pain.

## 2 Results

### 2.1 Optogenetic Activation of Midbrain Microglia Induces Anxiety‐ and Depression‐Like Behaviors

To model neuropathic pain–associated affective changes, we used the spared nerve injury (SNI) paradigm and established the behavioral timeline (Figure S1A). SNI produced robust mechanical allodynia from 1 to 10 weeks after surgery (Figure S1B). Mice subjected to SNI for 6 weeks also exhibited anxiety‐ and depression‐like behaviors, including decreased center entries and reduced time spent in the center zone during the open‐field test (OFT) (Figure S1C,D), increased immobility in the tail‐suspension test (TST) and forced swim test (FST) (Figure S1E,F), and diminished sucrose preference (Figure S1G). These data indicate that SNI induces both sensory hypersensitivity and negative affective states.

**FIGURE 1:**
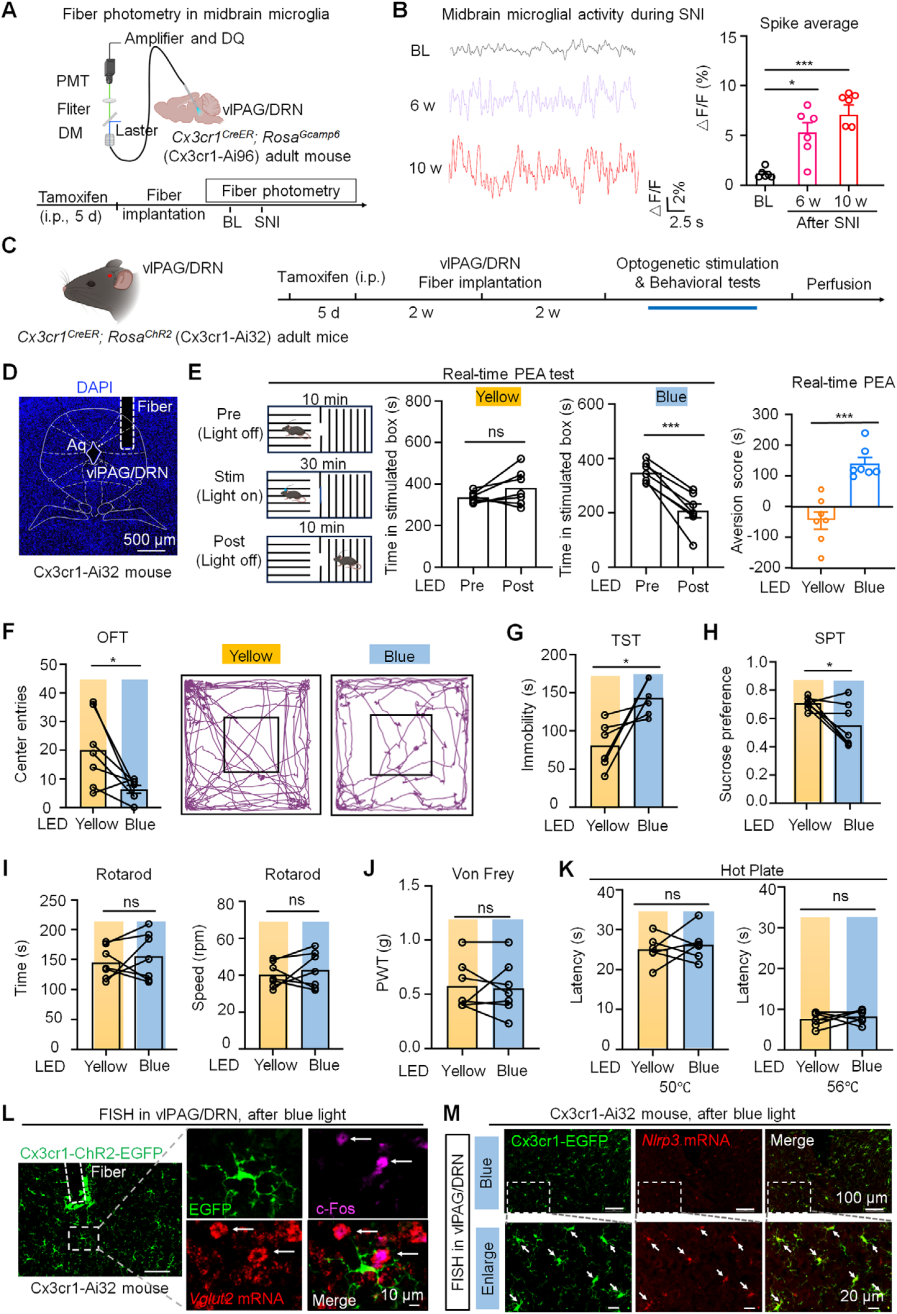
Optogenetic activation of midbrain microglia induces anxiety‐ and depression‐like behaviors. (A) Schematic of fiber photometry recording from microglia in the vlPAG/DRN of a *Cx3cr1^GCaMP6^; Rosa^CaMP6^* (Cx3cr1‐Ai96) mouse. (B) Representative vlPAG/DRN microglial activity traces at baseline (BL), 6 weeks (6 w), and 10 weeks after SNI (left), with quantification of spike average (right). Each dot represents one mouse (n = 6). (C) Experimental design for optogenetic behavioral tests in *Cx3cr1^CreER^; Rosa^ChR2^* (Cx3cr1‐Ai32) adult mice. (D) Representative image of optic fiber placement in the vlPAG/DRN of a Cx3cr1‐Ai32 mouse. Scale bar, 500 µm. (E) Real‐time place‐escape/avoidance (PEA) assay: protocol schematic (left), time spent in the light‐paired (stimulated) box before (Pre) and after (Post) yellow‐ or blue‐light stimulation (middle), and quantification of aversion score (right)(n = 7). (F) Open‐field test (OFT) showing center entries (left) and representative locomotion trajectories (right) under yellow‐ and blue‐light conditions (n = 7). (G–H) Tail‐suspension test (TST) (G) and sucrose‐preference test (SPT) (H) for depression‐like behaviors following midbrain microglial activation (n = 6–7). (I) Rotarod test assessing motor coordination and speed under yellow‐ and blue‐light conditions (n = 7). (J,K) Assessment of sensory thresholds, including mechanical (J, von Frey) and thermal sensitivity (K, hot plate test) (n = 6–7). (L) Representative fluorescent in situ hybridization (FISH) images showing the optic fiber tract and EGFP‐labeled microglia (left), and a magnified view of the boxed area (right) following optogenetic activation. Arrows indicate c‐Fos^+^ Vglut2^+^ (by FISH) neurons (magenta/red) adjacent to EGFP^+^ microglia (green). Scale bars, 100 µm (left) and 10 µm (right). (M) Co‐localization of *Nlrp3* mRNA (by FISH) with EGFP^+^ microglia in the vlPAG/DRN of a Cx3cr1‐Ai32 mouse following optogenetic activation. Scale bars, 100 µm (top) and 20 µm (bottom). ns, not significant. ^*^ *p* < 0.05, ^**^ *p* < 0.01, ^***^ *p* < 0.001, ^****^ *p* < 0.0001. Data in (B) were analyzed by one‐way ANOVA with Bonferroni post hoc test; data in (E–K) were analyzed by unpaired *t*‐test. Data are presented as mean ± SEM.

A previous study has implicated the ventrolateral periaqueductal gray–dorsal raphe nucleus (vlPAG/DRN) complex as a potential midbrain node that integrates nociceptive processing with affective regulation [22]. We therefore examined whether SNI recruits microglial activation in the vlPAG/DRN. Using long‐term fiber photometry in *Cx3cr1* ^CreER^; *Rosa^Gcamp6^* (Cx3cr1‐Ai96) mice implanted with an optical fiber targeting the vlPAG/DRN (Figure 1A), we observed a progressive increase in microglial calcium spikes at 6 and 10 weeks after SNI (Figure 1B). This increase coincided with depressive‐like behavior (Figure S1E–G), indicating persistent microglial hyperactivity in this region. Consistent with this, quantitative analysis revealed that SNI significantly increased the number of Iba1^+^ microglia and induced morphological activation in the vlPAG/DRN compared with sham controls (Figure S2A–F).

To test the sufficiency of microglial activation in a site‐specific manner, we optogenetically stimulated microglia in the vlPAG/DRN of *Cx3cr1* ^CreER^; *Rosa^ChR2^* (Cx3cr1‐Ai32) mice with blue light (470 nm), using yellow light (595 nm) as a control (Figure 1C,D). A single 30 min session of blue light stimulation induced immediate aversive‑like behaviors: mice exhibited avoidance of the stimulation‐paired chamber in a real‐time place escape/avoidance (PEA) assay (Figure 1E), reduced exploration of the center in the OFT (Figure 1F), increased immobility in the TST (Figure 1G), and decreased sucrose preference (Figure 1H). The stimulation did not alter motor performance on the rotarod, mechanical thresholds (von Frey test), or thermal sensitivity (hot‐plate test) (Figure 1I–K). Notably, fluorescent in situ hybridization (FISH) combined with immunostaining revealed that blue‐light stimulation robustly recruited nearby excitatory neurons, as evidenced by c‐Fos induction in VGLUT2^+^ neurons adjacent to EGFP^+^ (ChR2‐expressing) microglia (Figure 1L). Consistent with engagement of an inflammatory program, *Nlrp3* mRNA was detected specifically in EGFP^+^ microglia (Figure 1M).

### 2.2 Chemogenetic Activation of Midbrain Microglia Induces Anxiety/Depression‐Like Behaviors and Upregulates NLRP3 Signaling

**FIGURE 2:**
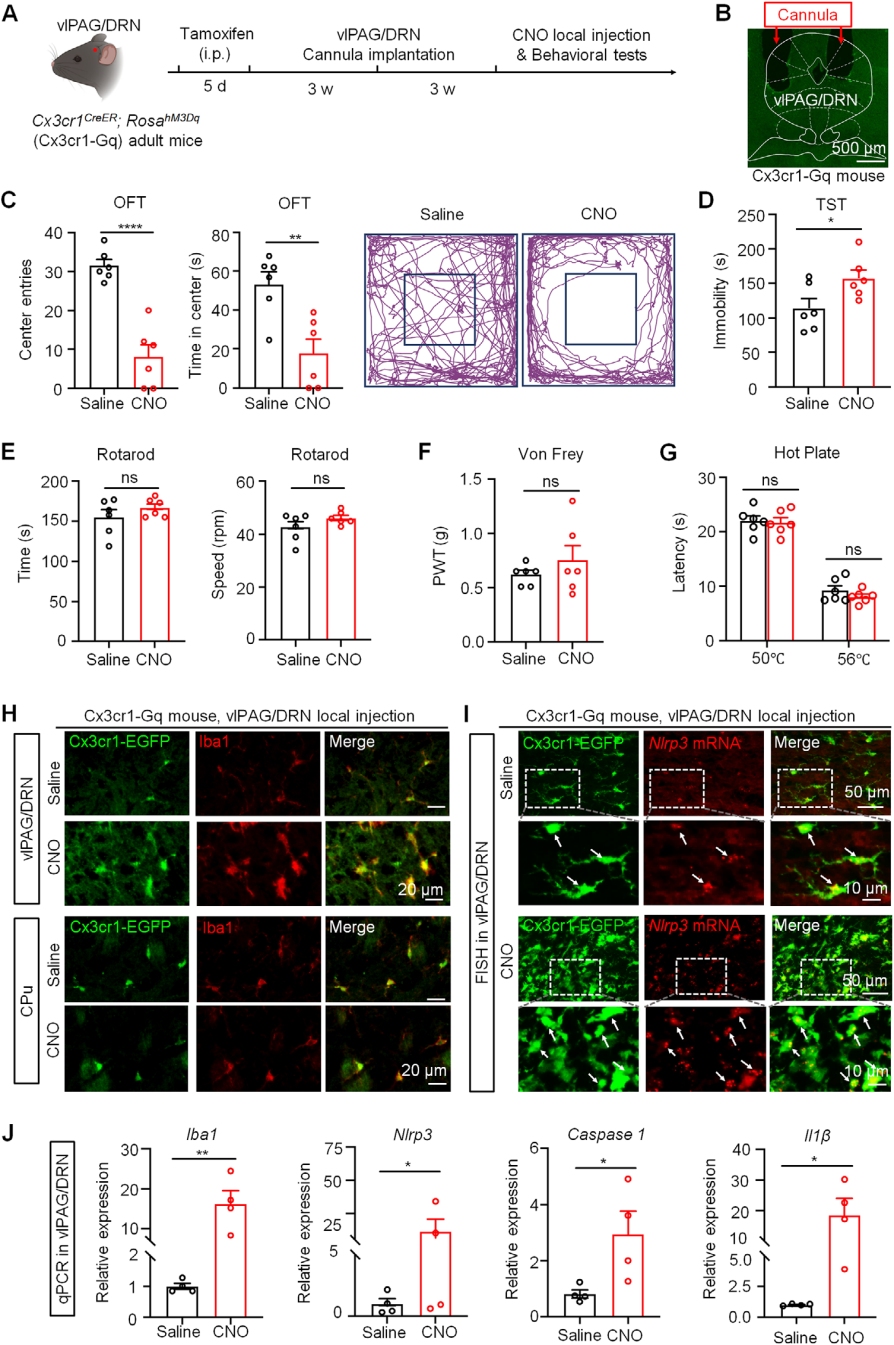
Chemogenetic activation of midbrain microglia induces anxiety/depression‐like behaviors and upregulates NLRP3 signaling. (A) Experimental design for chemogenetic behavioral tests in *Cx3cr1^CreER^; Rosa^hM3Dq^* (Cx3cr1‐Gq) mice. (B) Representative image showing guide cannula placement in the vlPAG/DRN of a Cx3cr1‐Gq mouse. Scale bar, 500 µm. (C) Open‐field test (OFT) showing center entries (left), time in center (middle), and representative locomotor trajectories (right) (saline, n = 6; CNO, n = 6). (D) Tail‐suspension test (TST) assessing depression‐like behavior following chemogenetic activation of midbrain microglia (saline, n = 6; CNO, n = 6). (E–G) Behavioral assessments of motor coordination (E, rotarod test), mechanical sensitivity (F, von Frey test), and thermal sensitivity (G, hot plate test) (saline, n = 6; CNO, n = 6). (H) Immunofluorescence images showing co‐localization of EGFP (hM3Dq) and Iba1^+^ microglia in the vlPAG/DRN and caudate putamen (CPu) of saline‐ and CNO‐treated mice. Scale bar, 20 µm. (I) Representative fluorescent in situ hybridization (FISH) images showing upregulated *Nlrp3* mRNA expression in EGFP^+^ microglia after local CNO treatment in the vlPAG/DRN. Scale bars, 50 µm (top) and 10 µm (bottom). (J) Quantitative PCR (qPCR) analysis of *Iba1*, *Nlrp3*, *Caspase 1*, and *Il1β* mRNA levels in the vlPAG/DRN of saline‐ and CNO‐treated mice (saline, n = 4; CNO, n = 4). ns, not significant. ^*^ *p* < 0.05, ^**^ *p* < 0.01, ^****^ *p* < 0.0001 by unpaired *t*‐test. Data are presented as mean ± SEM.

We next used a chemogenetic approach in *Cx3cr1* ^CreER^; *Rosa^hM3Dq^* (Cx3cr1‐Gq) mice (Figure 2A,B). Chemogenetic activation of vlPAG/DRN microglia reduced center exploration in the OFT (Figure 2C) and increased immobility in the TST (Figure 2D), without affecting motor coordination on the rotarod or mechanical and thermal nociceptive thresholds (Figure 2E–G). To exclude off‐target effects of the designer drug CNO, we compared microglial activation in the vlPAG/DRN and caudate putamen (CPu). Following CNO administration, microglial activation was evident only in the vlPAG/DRN (Figure 2H), arguing against appreciable spread of CNO to the CPu. Quantitative analysis revealed that chemogenetic activation significantly increased the number of EGFP^+^ microglia in the vlPAG/DRN and enlarged the somatic area of individual microglia (Figure S3A–D). Sholl analysis further revealed a reduction in microglial process complexity after CNO treatment, as indicated by fewer intersections across increasing distances from the soma and a significant decrease in total Sholl intersections (Figure S3E,F). Interestingly, chemogenetic activation recapitulated key morphological features of microglial activation observed under neuropathic pain conditions (Figure S2). In the vlPAG/DRN, CNO treatment upregulated *Nlrp3* mRNA in EGFP^+^ microglia (Figure 2I) and increased transcript levels of *Iba1*, *Nlrp3*, *Caspase 1*, and *Il1β* in tissue samples (Figure 2J).

Together, across both optogenetic and chemogenetic approaches, selective activation of vlPAG/DRN microglia was sufficient to elicit aversion as well as anxiety‐ and depression‐like behaviors, without altering locomotion or nociceptive thresholds. These results point to a local microglial NLRP3–IL‐1β pathway that engages nearby VGLUT2^+^ glutamatergic neurons, providing an upstream link between midbrain inflammation and negative affect.

### 2.3 Microglial NLRP3 Is Required for Anxiety‐ and Depression‐Like Behaviors Induced by Neuropathic Pain

**FIGURE 3:**
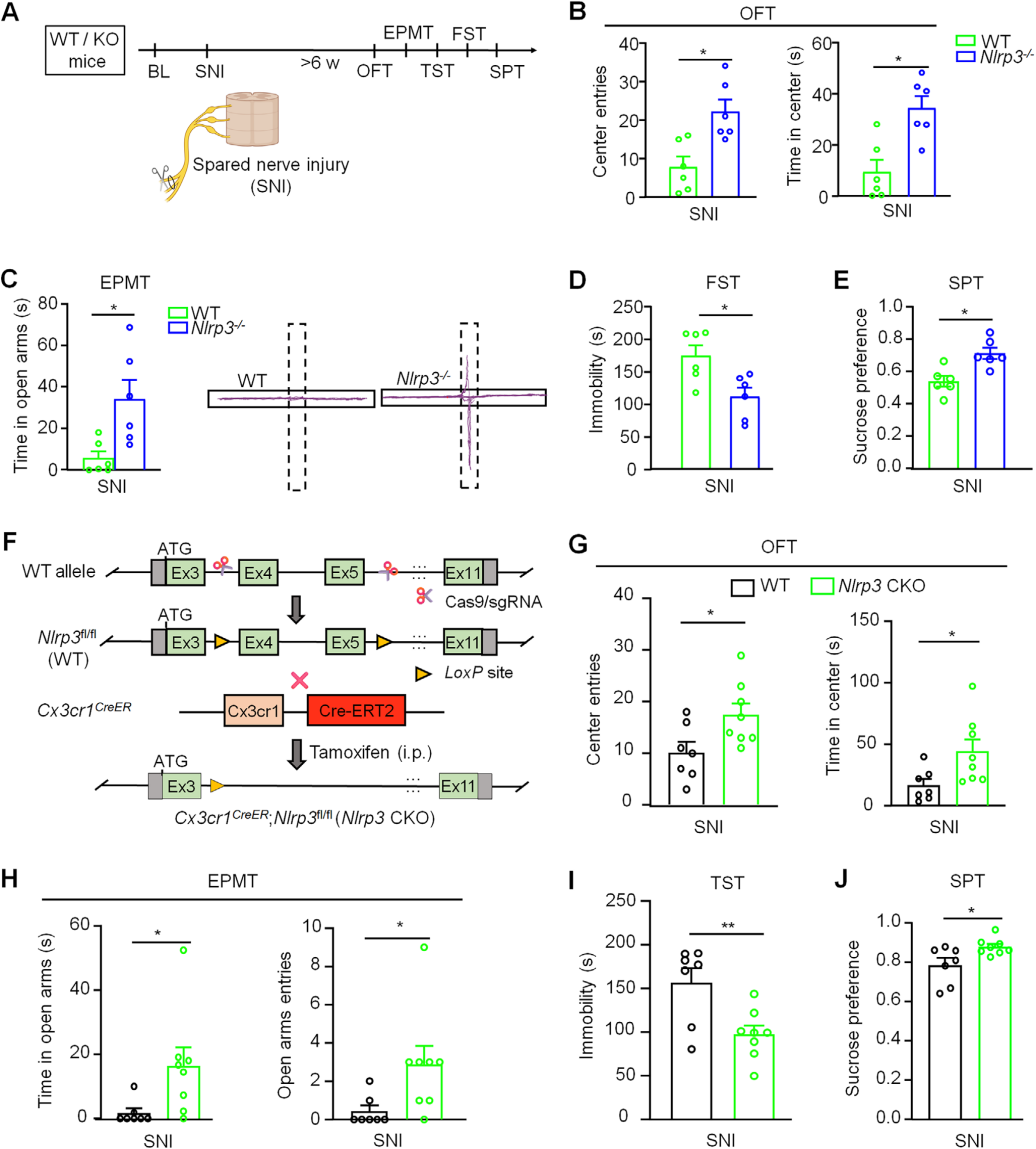
Microglial NLRP3 is required for anxiety‐ and depression‐like behaviors induced by neuropathic pain. (A) Experimental design for behavioral assays in WT and *Nlrp* ^3 − / −^ mice subjected to spared nerve injury (SNI). (B,C) Anxiety‐like behaviors assessed by the open‐field test (OFT) (B) and elevated plus maze test (EPMT) (C) in WT and *Nlrp* ^3 − / −^ mice after *SNI*(*WT*,  *n* = 6; *Nlrp* ^3 − / −^, *n* = 6). (D,E) Depression‐like behaviors assessed by the forced swim test (FST) (D) and sucrose preference test (SPT) (E) in WT and *Nlrp* ^3 − / −^ mice after SNI. (F) Schematic for generating microglia‐specific *Nlrp3* conditional knockout (CKO) mice. (G–J) Behavioral performance of WT and *Nlrp3* CKO mice after SNI in the OFT (G), EPMT (H), TST (I), and SPT (J) (WT, n = 7; *Nlrp3* CKO, n = 8). ^*^ *p* < 0.05, ^**^ *p* < 0.01; by unpaired *t*‐test. Data are presented as mean ± SEM.

The NLRP3 inflammasome promotes caspase‐1–dependent maturation of IL‐1β and has been linked to microglia‐driven neuroinflammation and mood dysregulation [23]. We hypothesized that NLRP3 contributes to the affective component of neuropathic pain. Global *Nlrp3* knockout (*Nlrp* ^3 − / −^) mice exhibited normal baseline physiology and sensorimotor performance (Figure S4A–F), indicating that any post‐injury behavioral benefit would reflect mitigation of negative affect rather than altered locomotion or nociception. Following SNI, *Nlrp* ^3 − / −^ mice displayed a markedly attenuated affective phenotype compared with wild‐type (WT) littermates, with improved exploratory behavior and reduced despair‐like readouts (Figure 3A–E).

Within the vlPAG/DRN, FISH combined with Iba1 immunostaining revealed *Nlrp3* mRNA signal in microglia that was increased after SNI (Figure S5A). Consistent with this, qPCR analysis of vlPAG/DRN tissue showed that SNI elevated transcript levels of *Nlrp3* and its downstream inflammatory targets (*Caspase 1*, *Il1β*, *Il18*) in WT mice, whereas this induction was abolished in *Nlrp* ^3 − / −^ mice (Figure S5B). These observations nominate a vlPAG/DRN microglial NLRP3–IL‐1β axis as a candidate link between injury and negative affect.

To test causality at the cellular level, we generated microglia‑specific conditional knockout mice (*Cx3cr1^CreER^*; *Nlrp3* ^fl/fl^, Figure 3F). Conditional deletion, like the global knockout, did not disrupt baseline physiology or affective‐related behaviors (Figures S4F–L), ruling out nonspecific behavioral confounds. After SNI, however, mice lacking *Nlrp3* specifically in microglia showed a substantially reduced negative‐affect phenotype, with restored exploratory behavior and normalized anxiety‐ and depression‐like measures (Figure 3G–J). Together, these data identify microglial NLRP3—with the vlPAG/DRN as a key site of injury‐evoked *Nlrp3* upregulation and downstream signaling—as a critical driver of neuropathic pain‐associated negative affect.

### 2.4 IL‐1β/IL‐1R1 Signaling Engages vlPAG/DRN VGLUT2^+^ Neurons to Drive Anxiety‐ and Depression‐Like Behaviors

**FIGURE 4:**
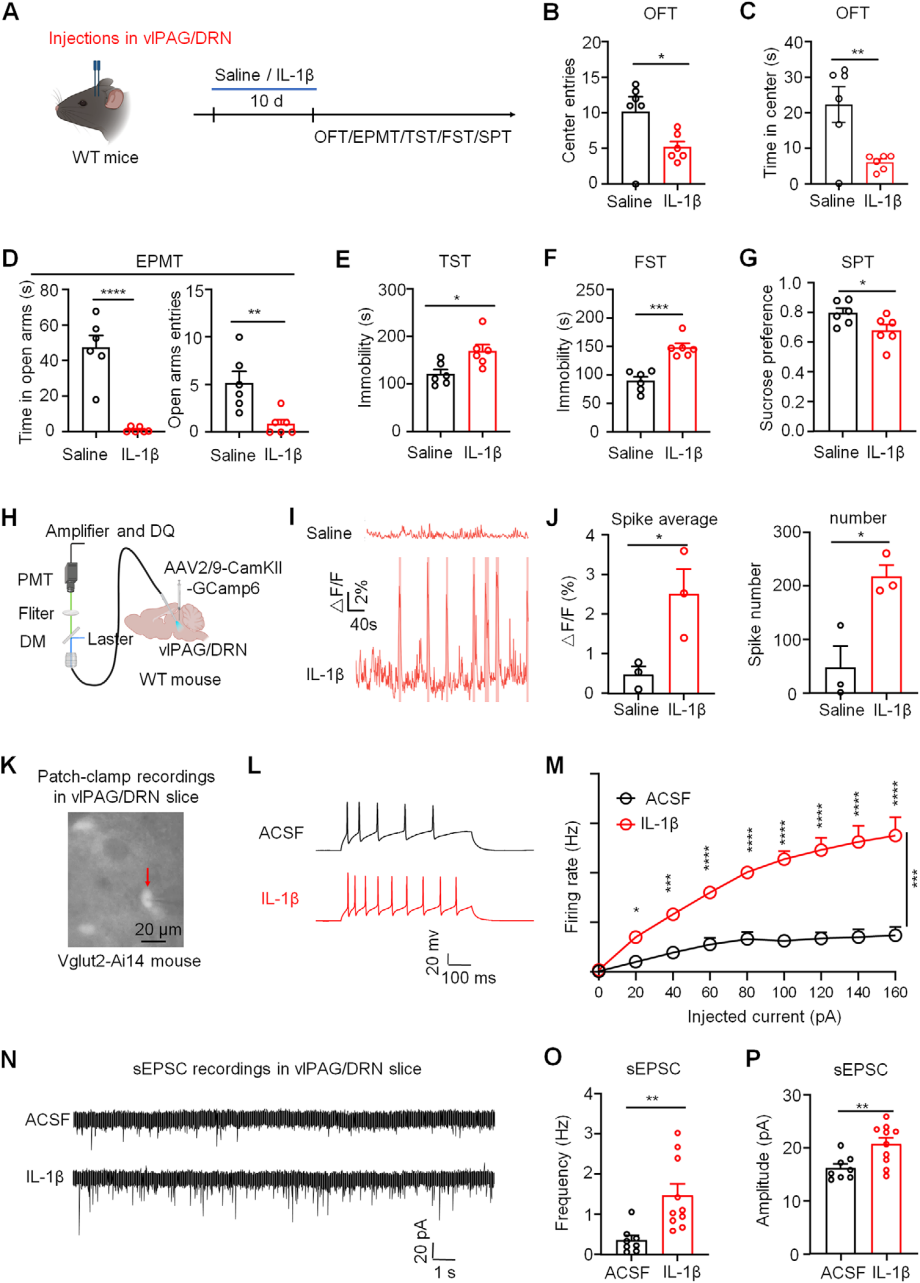
IL‐1β induces anxiety‐ and depression‐like behaviors by activating vlPAG/DRN^Glu^ neurons. (A) Experimental timeline for intra‐vlPAG/DRN injections of saline or IL‐1β in WT mice followed by behavioral testing. (B‐D) Anxiety‐like behaviors assessed by the open‐field test (OFT) (B,C) and elevated plus maze test (EPMT) (D) after saline or IL‐1β treatment (saline, n = 6; IL‐1β, n = 6). (E–G) Depression‐like behaviors assessed by the tail‐suspension test (TST) (E), forced swim test (FST) (F), and sucrose preference test (SPT) (G) after saline or IL‐1β treatment (saline, n = 6; IL‐1β, n = 6). (H) Schematic of fiber photometry recordings from vlPAG/DRN excitatory neurons expressing GCaMP6 in WT mice. (I and J) Representative ΔF/F traces (I) and quantification (J) of calcium spike average (left) and spike number (right) after saline or IL‐1β administration. Each dot represents one mouse (saline, n = 3; IL‐1β, n = 3). (K) Representative image showing a Vglut2^+^ neuron (red arrow) with whole‐cell patch‐clamp recording in a vlPAG/DRN slice from a Vglut2‐Ai14 mouse. (L and M) Representative action potential (AP) firing responses (L) and quantification of AP firing rate (M) of Vglut2^+^ neurons with ACSF or IL‐1β treatment (ACSF, n = 8; IL‐1β, n = 8). (N–P) Representative sEPSC traces (N) and quantification of sEPSC frequency (O) and amplitude (P) recorded from vlPAG/DRN Vglut2^+^ neurons with ACSF or IL‐1β treatment (ACSF, n = 8; IL‐1β, n = 10). ns, not significant. ^*^ *p* < 0.05, ^**^ *p* < 0.01, ^***^ *p* < 0.001, ^****^ *p* < 0.0001. Data in (B–G), (J), (O), and (P) were analyzed by unpaired *t*‐test; data in (M) were analyzed by two‐way ANOVA with Bonferroni's post hoc test. Data are presented as mean ± SEM.

To test sufficiency, focal delivery of recombinant IL‐1β into the vlPAG/DRN of naïve mice was sufficient to induce a robust negative‐affect phenotype—increasing anxiety‐ and depression‐like behaviors without affecting locomotion (Figure 4A). This was reflected by reduced exploratory drive in the open‐field and elevated plus‐maze tests, greater immobility in the tail‐suspension and forced swim tests, and lower sucrose preference (Figure 4B–G). In vivo fiber photometry further showed that IL‐1β increased calcium activity in vlPAG/DRN neurons (Figure 4H–J). In line with this, slice electrophysiology demonstrated that IL‐1β enhanced the excitability of vlPAG/DRN VGLUT2^+^ neurons, as evidenced by elevated firing responses (Figure 4K–M) and increased excitatory synaptic drive (higher sEPSC frequency and amplitude; Figure 4N–P), functionally linking IL‐1β signaling to recruitment of this neuronal population.

**FIGURE 5:**
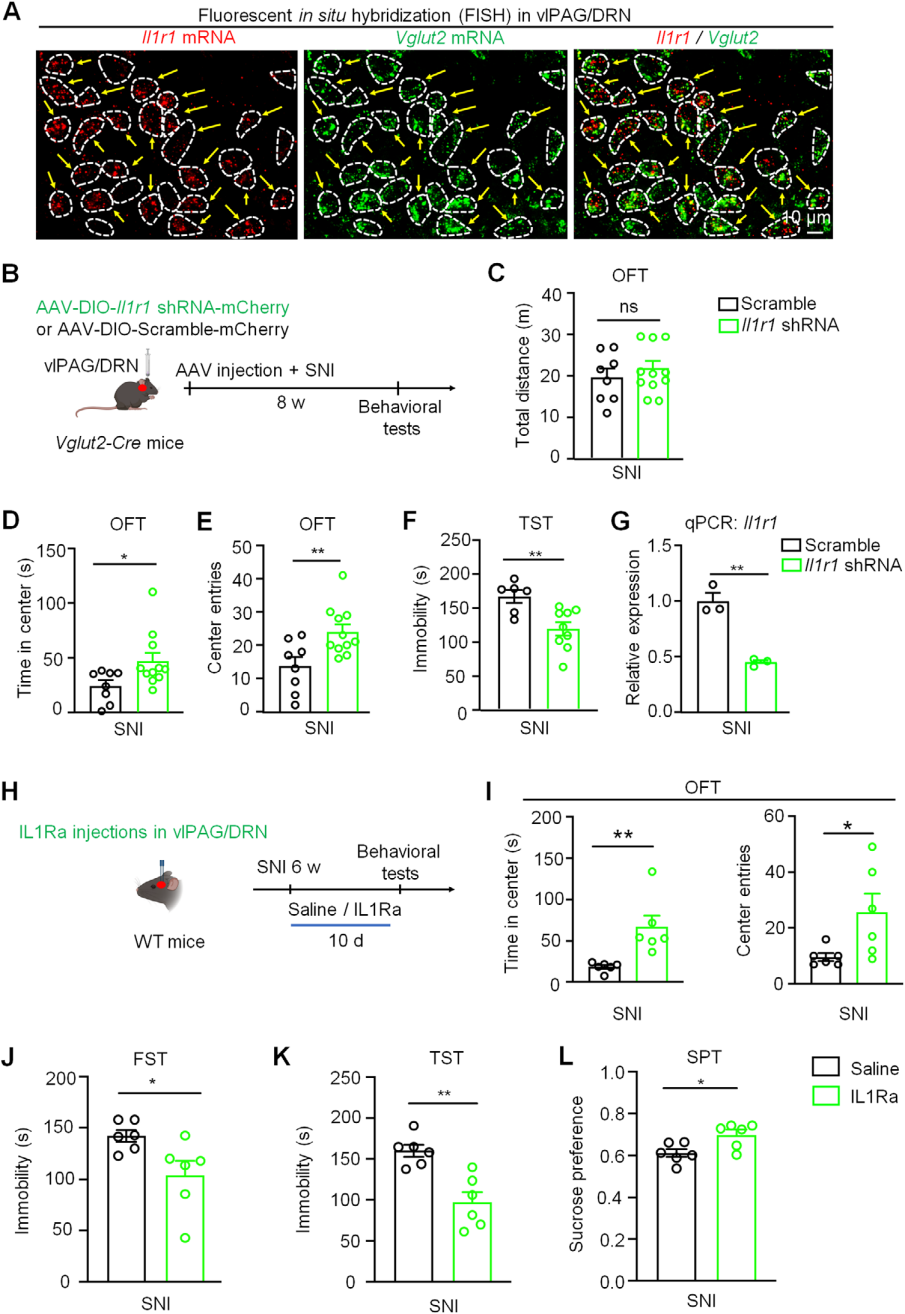
IL‐1R1 signaling in vlPAG/DRN^Glu^ neurons is essential for SNI‐induced anxiety‐ and depression‐like behaviors. (A) Representative fluorescent in situ hybridization (FISH) images showing *Il1r1* and *Vglut2* mRNA co‐expression in the vlPAG/DRN. Scale bars, 10 µm. (B) Experimental design for *Il1r1* knockdown in vlPAG/DRN Vglut2^+^ neurons and subsequent behavioral testing. (C–E) Open‐field test (OFT) assessing total distance traveled (C), time spent in the center (D), and number of center entries (E) in SNI mice expressing *Il1r1* shRNA or a scramble control (scramble, n = 8–9; *Il1r1* shRNA, n = 11). (F) Tail‐suspension test (TST) in SNI mice expressing *Il1r1* shRNA or a scramble control (scramble, n = 6; *Il1r1* shRNA, n = 9). (G) qPCR validation of *Il1r1* knockdown in vlPAG/DRN tissues (Scramble, n = 3; *Il1r1* shRNA, n = 3). (H) Experimental timeline for intra‐vlPAG/DRN administration of saline or IL‐1Ra (receptor antagonist) in SNI mice followed by behavioral testing. (I) Anxiety‐like behavior assessed by the open‐field test (OFT) showing that IL‐1Ra treatment alleviates SNI‐induced deficits (saline, n = 6; IL‐1Ra, n = 6). (J‐L) Depression‐like behaviors assessed by the forced swim test (FST, J), tail suspension test (TST, K), and sucrose preference test (SPT, L) showing that IL‐1Ra treatment alleviates SNI‐induced deficits (saline, n = 6; IL‐1Ra, n = 6). ns, not significant. ^*^ *p* < 0.05, ^**^ *p* < 0.01. Data were analyzed by unpaired *t*‐test and are presented as mean ± SEM.

In situ hybridization revealed co‐localization of *Il1r1* and *Vglut2* mRNA in the vlPAG/DRN, indicating that local VGLUT2^+^ neurons are positioned to directly sense IL‐1β (Figure 5A). To test whether IL‐1R1 signaling is required for the phenotype, we knocked down *Il1r1* in vlPAG/DRN VGLUT2^+^ neurons using AAV‐DIO‐*Il1r1* shRNA in *Vglut2‐Cre* mice (Figure 5B). Under SNI conditions, *Il1r1* knockdown ameliorated affective deficits without altering total locomotor activity (Figure 5C–F), and efficient knockdown of *Il1r1* was confirmed by qPCR (Figure 5G). Similarly, local blockade of IL‐1β signaling with the IL‐1R1 receptor antagonist (IL‐1Ra) in the vlPAG/DRN rescued exploratory behavior and normalized affective readouts in SNI mice (Figure 5H–L). These results indicate that IL‐1R1 signaling in the vlPAG/DRN is required for the expression of pain‐associated negative affect.

We next asked whether vlPAG/DRN VGLUT2^+^ neurons themselves are both sufficient and necessary to drive this state. Chemogenetic activation of these neurons with an ultra‐low dose (0.01 mg/kg) of CNO recapitulated the anxiety‐ and depression‐like profile (Figure S6A–F) without affecting locomotion (total distance in the OFT) or thermal nociception (Figure S6G). Conversely, chemogenetic inhibition of these neurons in SNI mice alleviated the behavioral abnormalities (Figure S6H–K). Together, these data identify vlPAG/DRN VGLUT2^+^ neurons as the critical IL‐1R1–responsive effectors that link local inflammatory signaling to negative affect.

### 2.5 The vlPAG/DRN^Glu^–BNST Circuit Drives Negative Affect in Neuropathic Pain

**FIGURE 6:**
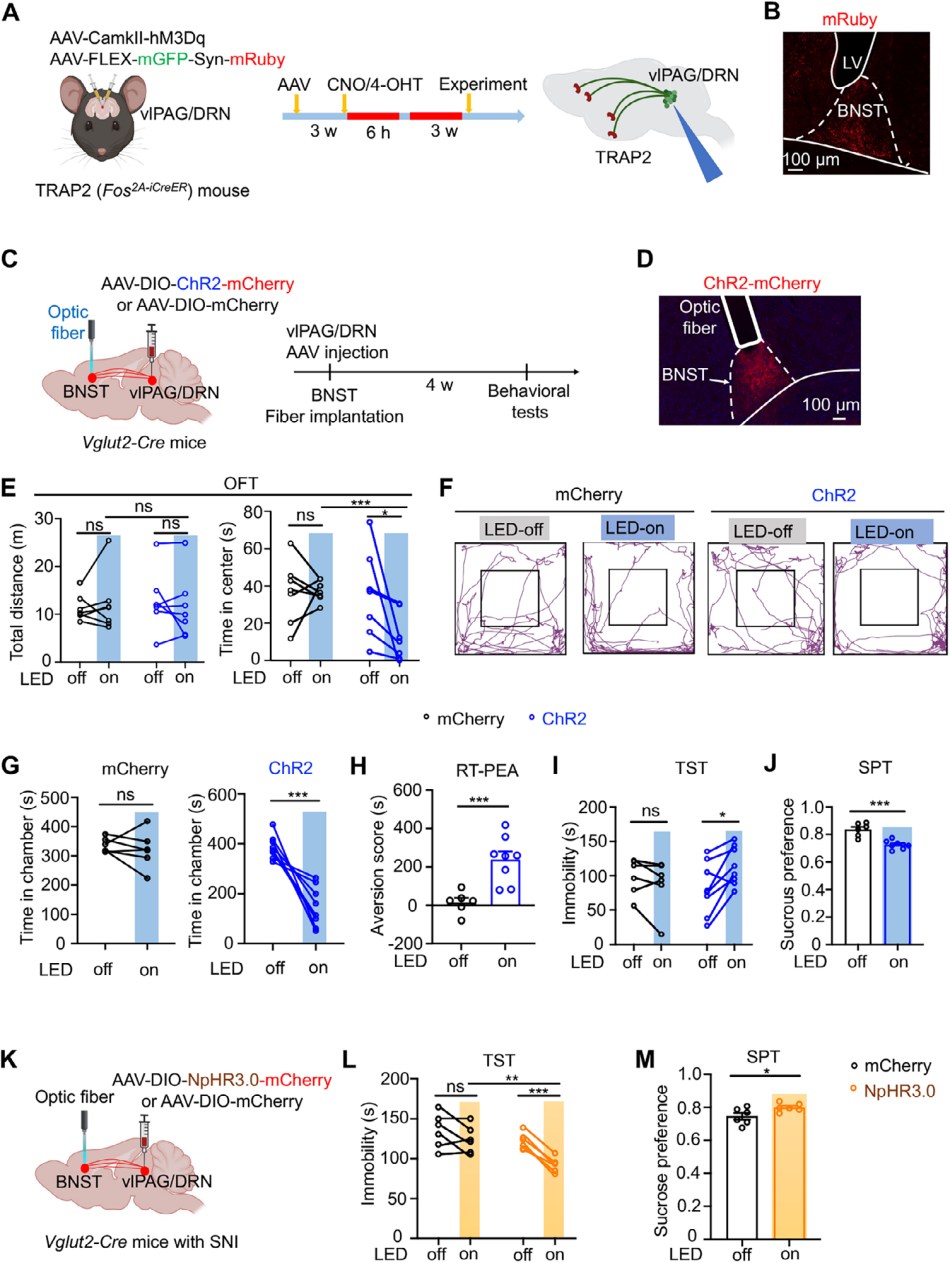
Activation of vlPAG/DRN^Glu^‐BNST circuit induces anxiety/depression‐like behaviors and is required for neuropathic pain‐induced depressive behaviors. (A) Experimental design in TRAP2 (*Fos^2A‐iCreER^*) mice. (B) Representative image showing mRuby expression in the BNST. Scale bars, 100 µm. (C) Viral strategy for selective activation of the vIPAG/DRN^Glu^ → BNST circuit for experiments in (E–J). (D) Representative fluorescence image showing ChR2‐mCherry expression and fiber placement in the BNST. Scale bars, 100 µm. (E and F) Open‐field test (OFT) under LED‐off and LED‐on conditions in mice expressing mCherry or ChR2‐mCherry, showing time in the center (E) and representative locomotion trajectories (F) (mCherry, n = 7; ChR2, n = 7). (G,H) Real‐time place escape/avoidance (PEA) test under LED‐off and LED‐on conditions (G) and quantification of aversion score (H) (mCherry, n = 6; ChR2, n = 8). (I and J) Tail‐suspension test (TST) (I) and sucrose preference test (SPT) (J) following optogenetic activation of vlPAG/DRN^Glu^–BNST projections (mCherry, n = 6; ChR2, n = 8). (K) Viral strategy for selective inhibition of the vlPAG/DRN^Glu^ → BNST circuit for the experiments in (L,M). (L,M) Tail‐suspension test (TST) (L) and sucrose preference test (SPT) (M) under LED‐off and LED‐on conditions following optogenetic inhibition of the circuit in SNI mice (mCherry, n = 6; NpHR4.0, n = 6). ns, not significant. ^*^ *p* < 0.05, ^**^ *p* < 0.01,^***^ *p* < 0.001. Data in (E), (G), (I), and (L) were analyzed by two‐way repeated‐measures (RM) ANOVA with post hoc test; data in (H), (J), and (M) were analyzed by unpaired *t*‐test. Data are presented as mean ± SEM.

Having established the necessity of vlPAG/DRN glutamatergic neurons (vlPAG/DRN^Glu^), we mapped their outputs. Using TRAP2 (*Fos^2A‐iCreER^*) mice [24] to capture neurons engaged by brief chemogenetic activation (Figure 6A), we observed dense axonal labeling in the BNST (Figure 6B), nominating it as a principal target. To test function, we expressed ChR2 in the vlPAG/DRN of *Vglut2‐Cre* mice and stimulated their terminals in the BNST (Figure 6C,D). This optogenetic activation reduced open‐field center time without altering total locomotion (Figure 6E; trajectories in Figure 6F), produced robust real‐time place aversion (Figure 6G,H), increased immobility in the tail‐suspension test (Figure 6I), and lowered sucrose preference (Figure 6J), indicating this projection is sufficient to drive aversive, anxiety‐like, and depression‐like behaviors. Conversely, in SNI mice, optogenetic inhibition of the same terminals with NpHR3.0 attenuated despair‐like behavior and restored reward‐related behavior (Figure 6K–M), demonstrating the necessity of this pathway for pain‐associated affective deficits.

**FIGURE 7:**
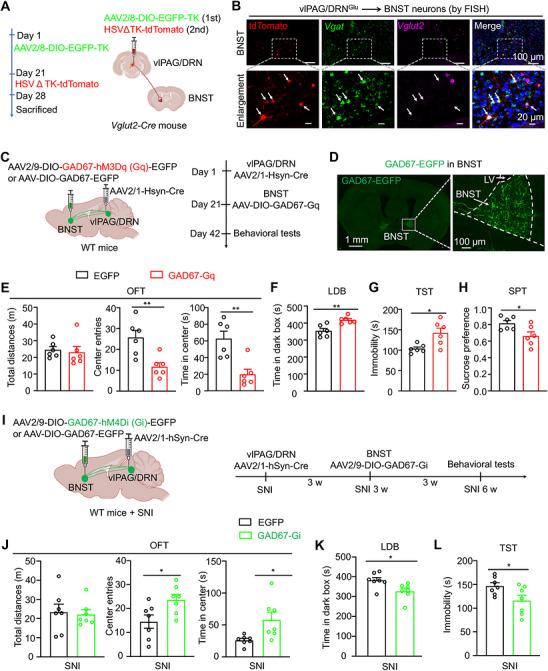
BNST GABAergic neurons are critical for anxiety‐ and depression‐like behavior in SNI mice. (A) Viral strategy for HSV‐based monosynaptic anterograde labeling of BNST neurons from vlPAG/DRN^Glu^ inputs in a *Vglut2*‐Cre mouse. (B) Representative FISH images in the BNST showing tdTomato^+^ neurons (labeled from vlPAG/DRN VGLUT2^+^ projections) co‐expressing Vgat mRNA (green) but not Vglut2 mRNA (magenta). Enlarged views are shown below. Scale bars, 100 µm (top) and 20 µm (bottom). (C) Viral strategy for chemogenetic activation of BNST GABA neurons receiving vlPAG/DRN VGLUT2^+^ input for experiments in (E–H). (D) Representative fluorescence images showing GAD67‐EGFP expression in the BNST. Scale bars, 1 mm (left) and 100 µm (right). (E,F) Anxiety‐like behaviors assessed by the open‐field test (OFT) (E) and light‐dark box test (LDB) (F) following chemogenetic activation (EGFP, n = 6; GAD67‐Gq, n = 6). (G and H) Depression‐like behaviors assessed by the tail‐suspension test (TST) (G) and sucrose preference test (SPT) (H) following chemogenetic activation (EGFP, n = 6; GAD67‐Gq, n = 6). (I) Viral strategy for chemogenetic inhibition of BNST GABA neurons receiving vlPAG/DRN VGLUT2^+^ input in SNI mice for experiments in (J–L). (J,K) Anxiety‐like behaviors assessed by OFT (J) and LDB (K) following chemogenetic inhibition in SNI mice (EGFP, n = 7; GAD67‐Gi, n = 7). (L) Depression‐like behavior assessed by TST (L) following chemogenetic inhibition in SNI mice (EGFP, n = 7; GAD67‐hM4Di, n = 7). ns, not significant. ^*^ *p* < 0.05, ^**^ *p* < 0.01,^***^ *p* < 0.001. Data were analyzed by unpaired *t*‐test and are presented as mean ± SEM.

We next sought to identify the BNST recipient population. An anterograde trans‐synaptic strategy (Figure 7A and Figure S7A,B) preferentially labeled BNST neurons postsynaptic to vlPAG/DRN VGLUT2^+^ inputs. These recipient cells co‐localized with *Vgat* mRNA but not *Vglut2* mRNA (mCherry^+^/Vgat^+^/Vglut2^−^; Figure 7B), identifying BNST GABAergic neurons (BNST^GABA^) as the principal downstream target. An anterograde Cre‐relay approach (AAV2/1‐hSyn‐Cre in vlPAG/DRN with AAV‐DIO‐GAD67‐hM3Dq‐EGFP in BNST; Figure 7C,D) confirmed that chemogenetic activation of this defined BNST GABAergic population recapitulated negative‐affect behaviors, including reduced open‐field center exploration, increased dark‐side occupancy in the light–dark box, elevated TST immobility, and reduced sucrose preference (Figure 7E–H). Conversely, inhibiting BNST GABAergic recipients in SNI mice rescued these affective measures (Figure 7I–L). In addition, retrograde labeling from the BNST with AAV‐Retro‐DIO‐mCherry robustly labeled somata in the vlPAG/DRN of *Vglut2‐Cre* mice, but yielded few labeled neurons in *Gad2‐Cre* mice (Figure S7C,D), confirming a predominantly glutamatergic origin.

Together, these convergent anatomical and functional data establish the vlPAG/DRN^Glu^→BNST^GABA^ circuit as a key downstream pathway that is both sufficient to induce and required to sustain negative‐affect behaviors in neuropathic pain.

## 3 Discussion

Neuropathic pain is frequently accompanied by debilitating anxiety and depression [25, 26, 27, 28, 29], yet the neuroimmune circuits that transform persistent nociception into negative affect remain poorly defined. Here, we identify a complete microglia–neuron–projection axis within the vlPAG/DRN that is necessary and sufficient to drive affective pathology. We show that neuropathic pain activates microglial NLRP3–IL‐1β signaling in this hub. IL‐1β then acts on local IL‐1R1‐expressing VGLUT2^+^ glutamatergic neurons, enhancing their excitability and driving a specific downstream projection to GABAergic neurons in the BNST (Figure S8). Silencing this vlPAG/DRN^Glu^→BNST^GABA^ pathway alleviates affective deficits in neuropathic pain mice. Our findings reveal how a peripheral injury can recruit a dedicated midbrain immune–glutamatergic circuit to impose a persistent negative‐valence state

### 3.1 Microglial NLRP3–IL‐1β Signaling Links Neuropathic Pain to Negative Affect

We found that nerve injury induces sustained microglial hyperactivity in the vlPAG/DRN and engages the NLRP3 inflammasome, a key driver of IL‐1β maturation [30, 31]. Both global and microglia‐specific deletion of *Nlrp3* attenuated the affective consequences of neuropathic pain without altering sensory thresholds or locomotion. This aligns with the established role of NLRP3 in neuroinflammation and mood dysregulation [32, 33]. Elevated IL‐1β is linked to depressive symptoms in patients and promotes despair‐like behavior in rodent models [34, 35].

In the vlPAG/DRN, SNI increased *Nlrp3* and *Il1β* expression, an effect that was abolished in Nlrp3^−^/^−^ mice. This positions microglial NLRP3–IL‐1β signaling as a critical inflammatory trigger within a key pain–affect integrator hub. Recent optogenetic work links microglial activation to increased ATP release events, raising the possibility that extracellular ATP dynamics may represent a tractable trigger for inflammasome engagement [36, 37]. Future work should identify the upstream injury signals (e.g., purinergic cues like ATP via P2×7/P2Y12 receptors) that engage NLRP3 in vlPAG/DRN microglia.

Although our data support the involvement of the canonical NLRP3–caspase‐1–IL‐1β pathway in vlPAG/DRN microglia after SNI, IL‐1β maturation may also involve caspase‐1‐independent proteases, such as neutrophil‐derived serine proteases, caspase‐8, MMP9, and granzyme A [38, 39, 40, 41]. Therefore, alternative IL‐1β‐processing pathways may also contribute under neuropathic pain conditions and should be examined in future studies.

### 3.2 A Microglia–IL‐1β–IL‐1R1 (VGLUT2^+^ Neuron) Interface Converts Neuroinflammation Into Pathological Affect

Under physiological conditions, microglia help maintain neuronal homeostasis and support synaptic function by shaping the local microenvironment around neurons [8, 42, 43, 44, 45, 46]. Under chronic challenge, however, microglia can undergo sustained activation and adopt a proinflammatory profile, releasing danger‐associated signals and cytokines such as IL‐1β. This maladaptive state has been implicated in diverse central nervous system disorders, including Parkinson's disease, Alzheimer's disease, and mood‐related pathology [7, 13, 47, 48].

Recent studies further suggest that microglia are not merely bystanders in depression‐like states but can act as active drivers of affective disturbance, making microglial signaling a potential therapeutic target for mood‐related symptoms [6, 49]. Consistent with this view, stress paradigms and chronic pain both increase microglial reactivity in midbrain structures, including the PAG [50]. We build on this by showing that neuropathic pain engages a microglial NLRP3–IL‐1β signaling cascade specifically within the vlPAG/DRN complex. We found that IL‐1β from activated microglia acts on nearby IL‐1R1‐expressing VGLUT2^+^ neurons, increasing their excitability and synaptic drive. Chemogenetic activation of these neurons was sufficient to induce robust anxiety‐ and depression‐like behaviors at stimulation levels that did not cause motor impairment [51]. Conversely, inhibiting these neurons alleviated affective deficits in neuropathic pain. This identifies a defined VGLUT2^+^ subpopulation as the critical effector that translates inflammatory signals into a persistent negative‐valence state. While our study focuses on IL‐1β, other cytokines (e.g., TNFα, IL‐6) likely contribute [52, 53]. Lastly, while we measured classic anxiety‐ and despair‐like endpoints, clinical depression encompasses broader phenotypes like anhedonia that remain to be explored in this circuit [54].

### 3.3 vlPAG/DRN Glutamatergic Neurons Encode Persistent Negative Valence

The vlPAG is a key regulator of nociceptive processing and defensive behavior. Targeted activation of VGLUT2^+^ glutamatergic neurons in the vlPAG produces robust analgesia and can also evoke freezing‐like defensive responses [15, 55]. These two behavioral outcomes appear to rely on partially segregated downstream projection pathways: descending vlPAG→rostral ventromedial medulla (RVM) output is associated with antinociception, whereas projections toward tectal/collicular targets support passive defensive freezing [15, 55]. In line with this, distinct vlPAG neuronal subpopulations can drive opposing affective states, with glutamatergic and GABAergic neurons biasing behavior toward either active coping/analgesia or passive defensive/aversive responses, respectively [15, 16]. Together, these observations indicate that the vlPAG/DRN region is not functionally uniform, but instead comprises multiple subcircuits that differentially shape pain modulation, fear expression, and affective state.

Building on this framework, we find that controlled activation of VGLUT2^+^ neurons within the vlPAG/DRN complex is sufficient to induce a persistent negative‐affective state. Consistent with prior work showing that stimulating vlPAG VGLUT2^+^ neurons can provoke anxiety‐like behavior [55], we observed that chemogenetic activation of these neurons elicited pronounced anxiety‐like and depression‐like behaviors. Critically, by titrating the CNO dose, we dissociated acute fear‐like immobility from sustained negative affect: higher‐level activation produced classic freezing‐like responses, whereas lower‐level activation that did not impair locomotion was sufficient to induce robust anxiety‐ and depression‐like phenotypes. Conversely, suppressing the activity of these vlPAG/DRN VGLUT2^+^ neurons after neuropathic pain (SNI) attenuated both anxiety‐like and depression‐like behaviors.

Taken together, these results argue that a defined subset of vlPAG/DRN VGLUT2^+^ neurons encodes a persistent negative‐valence signal rather than merely triggering an acute defensive freeze. In this view, the vlPAG/DRN complex functions not only as a rapid sensor‐effector node for analgesia and defensive reactions, but also as a central hub that stabilizes and maintains anxiety‐ and depression‐like states following chronic pain.

### 3.4 A vlPAG/DRN → BNST Pathway Broadcasts Negative Valence to the Forebrain

The vlPAG/DRN is embedded in a broader network of affect‐regulating circuits. Upstream inputs to this region have been shown to bidirectionally shape affective state [56]: for example, GABAergic projections from the medial preoptic area drive depressive‐like behavior following ovarian hormone withdrawal [16], whereas activation of a dmPFC→vlPAG glutamatergic pathway exerts antinociceptive and anxiolytic effects in neuropathic pain models [19]. By contrast, comparatively little was known about how vlPAG/DRN output influences downstream forebrain structures to maintain negative affect. Converging evidence suggests that the bed nucleus of the stria terminalis (BNST)—a stress‐ and threat‐responsive component of the extended amygdala enriched in GABAergic neurons [57, 58, 59]—acts as a hub for sustained anxiety‐like and depressive‐like states. Chronic pain, chronic stress, and substance withdrawal have all been shown to increase inhibitory drive within defined BNST microcircuits, including BNST projections to the ventral tegmental area (VTA) and lateral hypothalamus (LH), and this enhanced inhibition correlates with anxiety‐like and depression‐like behavioral phenotypes [4, 57, 60, 61, 62]. For example, neuropathic pain heightens the excitability of ovBNST neurons, and chemogenetic suppression of these neurons alleviates pain‐induced anxiety‐like behavior [4]. These findings support a model in which persistent negative affect arises, in part, from strengthened inhibitory control within BNST output pathways that regulate mesolimbic and hypothalamic targets.

Consistent with this forebrain framework, our results identify and functionally validate a specific midbrain output—a vlPAG/DRN→BNST pathway—that broadcasts negative valence and links microglia‐driven activity to forebrain affective bias. We found that selectively silencing the vlPAG/DRN VGLUT2^+^ → BNST GABA projection produced both anxiolytic and antidepressant‐like effects in SNI mice. This indicates that the persistent negative valence encoded by a subset of vlPAG/DRN VGLUT2^+^ neurons is not confined to local defensive or nociceptive control, but is broadcast to the BNST, where it can be stabilized as a chronic “threatened” internal state. These data suggest that neuropathic pain may recruit a midbrain‐to‐BNST aversive pathway that parallels, and potentially converges with, previously described BNST inhibitory motifs targeting the VTA and LH [4, 57, 60, 61, 62].

In this framework, microglial NLRP3–IL‐1β signaling in the vlPAG/DRN not only tunes local VGLUT2^+^ neuron excitability but also ultimately biases BNST output toward a durable negative affective mode.

### 3.5 Limitations of the Study

One limitation of this study is that all experiments were performed exclusively in male mice. This is particularly important because microglial mechanisms in chronic pain exhibit sex differences. Previous studies have demonstrated that neuropathic pain hypersensitivity in male mice depends more strongly on microglial signaling, whereas females may rely more on microglia‐independent or adaptive immune mechanisms [63, 64]. Sex‐specific microglial transcriptional responses after peripheral nerve injury have also been reported [65]. Thus, although our data identify a microglia‐dependent NLRP3–IL‐1β–VGLUT2^+^ pathway in the male vlPAG/DRN, whether this pathway operates similarly in females remains to be examined. Future studies including female mice are needed to determine the sex dependence of this neuroimmune circuit.

Another limitation is that, although we refer to the manipulated region as the vlPAG/DRN throughout this study, the vlPAG and DRN are anatomically adjacent but functionally distinct. The vlPAG is implicated in pain modulation, defensive responses, and anxiety‐related behaviors [66, 67], whereas the DRN regulates stress, mood, and affective behaviors through heterogeneous serotonergic and non‐serotonergic neurons [68, 69]. Because our viral, optogenetic, and pharmacological manipulations covered the vlPAG/DRN region, we cannot determine whether vlPAG or DRN microglia play a more dominant role. We speculate that vlPAG microglia may preferentially contribute to pain‐associated defensive and anxiety‐like responses, whereas DRN microglia may be more associated with stress‐ and mood‐related depressive‐like behaviors. Recent studies have also treated the adjacent vlPAG/DR or vlPAG‐DR region as a functionally interacting unit in pain and affective behaviors [70, 71]. Future studies using more spatially restricted manipulations will be required to define the distinct or cooperative roles of vlPAG and DRN microglia in this neuroimmune pathway.

## 4 Conclusion and Therapeutic Implications

In summary, our results demonstrate that chronic nociceptive input can be translated into persistent negative affect through a defined neuroimmune pathway: NLRP3 activation in vlPAG/DRN microglia drives IL‐1β release, which acts on local IL‐1R1–expressing VGLUT2^+^ neurons, driving their output to the BNST and thereby sustaining anxiety‐ and depression‐like states (Figure S8). These findings position the vlPAG/DRN not only as a nociceptive relay, but also as an inflammation‐sensitive affective hub; in neuropathic pain, its projection to the BNST helps consolidate a persistent threat state and negative emotional bias. By linking microglial inflammatory signaling to a projection‐defined glutamatergic subpopulation and to the BNST as a forebrain node, our work provides a concrete circuit‐level explanation for how chronic pain evolves into an affective disorder and highlights the vlPAG/DRN IL‐1β/IL‐1R1–VGLUT2^+^ neuron interface and the vlPAG/DRN→BNST pathway as potential therapeutic targets.

## 5 Experimental Section

### 5.1 Key Resources Table

Please refer to Table S1 for detailed information on antibodies, viral reagents, and other key resources used in the present study.

### 5.2 Animals

Details of all mouse strains are provided in Table S1. Mice were group‐housed under a 12 h light/dark cycle with ad libitum access to food and water, and bred in the animal facility of Zhejiang University. Adult male mice (8–12 weeks) were used for behavioral and pharmacological experiments. Animals were randomly assigned to different experimental groups. All animal procedures were conducted in accordance with the National Institutes of Health Guidelines and approved by the Animal Care and Use Committee of Zhejiang University.

### 5.3 The Spared Nerve Injury (SNI) Neuropathic Pain Model

Mice were anesthetized with isoflurane (induction: 4%–5%, maintenance: 1.5%–2%). An incision was made in the skin and thigh muscle of the left hindlimb to expose the sciatic nerve and its three branches (sural, common peroneal, and tibial nerves). The common peroneal and tibial nerves were tightly ligated with non‐absorbable 4‐0 chromic gut sutures and transected, removing ∼2 mm segments. The sural nerve was left intact. The wound was closed with sutures and disinfected with iodophor. Sham‐operated mice underwent the same surgical exposure without nerve ligation or transection.

### 5.4 Stereotaxic Surgery

Stereotaxic injections were performed bilaterally under isoflurane anesthesia. Glass micropipettes delivered virus at 50–100 nL/min (100–200 nL per site per hemisphere); the pipette was left in place for 5–10 min post‐infusion to minimize reflux. Coordinates were referenced to bregma, with depths measured from the skull surface:

vIPAG/DRM:AP−4.55mm,ML±0.55mm,DV−2.85mm

BNST:AP+.028mm,ML±1.14mm,DV−4.28mm

Unless otherwise noted, behavioral testing or imaging began 3–4 weeks after injection. Animals were randomly assigned to groups; behavioral scoring, image quantification, and statistical analyses were performed under blinded conditions. Pre‐specified exclusion criteria included mistargeted injections or implants, failed viral expression, fiber or cannula loss, and postoperative complications. Injection and implant locations were verified histologically at the end of each experiment.

### 5.5 In Vivo Optogenetics

Following viral delivery, an optical fiber (200 µm core; Inper) was positioned 150 µm above the injection site and permanently secured with Metabond (Parkell) and dental acrylic. Behavioral experiments were initiated 3–4 weeks later to allow expression. Light was delivered via a dual‐wavelength laser system (Aurora‐400‐470‐589, NEWDOON) coupled to the implanted fiber; output was calibrated at the fiber tip before each session. ChR2 activation was achieved with blue light (470 nm; 1–3 mW; 15 ms pulses; 20 Hz). eNpHR3.0 inhibition was achieved with yellow light (595 nm; 6–10 mW; constant). Optical stimulation for microglial activation was delivered at 10 Hz with a pulse width of 5 ms and a power of 5 mW. Each stimulation session lasted 30 min [72].

### 5.6 In Vivo Chemogenetics

CNO (MCE, #HY‐17366A) was administered intraperitoneally (i.p.) following a 3–4 weeks viral expression period, typically 30 min prior to behavioral assessment. For hM3Dq (Gq) activation, CNO was administered at 2 mg/kg in all cohorts except those targeting vlPAG/DRN glutamatergic neurons, where an ultra‐low dose (0.01 mg/kg) was used to avoid freezing. For hM4Di (Gi) inhibition, CNO was administered at 3–5 mg/kg. Behavioral testing was initiated 20–40 min after CNO administration. Vehicle controls received sterile saline or 0.5% DMSO in saline at matched volumes.

### 5.7 Intracranial Cannula Infusions

Bilateral guide cannulae (RWD) were implanted 200–300 µm above the target (vlPAG/DRN or BNST), anchored with skull screws and dental acrylic. Animals were allowed to recover for ≥7 days. For local delivery, an internal injector extending 0.5–1.0 mm beyond the guide was inserted, and drug was infused via a microsyringe pump at 50–100 nL/min to a total volume of 0.2–0.3 µL per side. The injector was left in place for 5–10 min post‐infusion to limit reflux. Drugs included IL‐1β (30 ng, R&D Systems, #201‐LB‐100/CF), IL‐1 receptor antagonist (IL‐1Ra, 100 ng, R&D Systems, #280‐RA‐050/CF), and CNO (0.3–1 mM), each dissolved in sterile saline. Behavioral assays were performed according to a pre‐specified sequence after infusion.

### 5.8 Activity‐Dependent Neuronal Labeling (TRAP2)

Fos^2A‐iCreER^ (TRAP2) mice were bilaterally injected in the vlPAG/DRN with a mixture of AAV‐CaMKIIα‐hM3Dq‐mCherry and AAV‐FLEX‐mGFP‐Syn‐mRuby. Following a ≥3‐week post‐injection recovery and viral expression period, mice were singly housed in a dark environment overnight prior to labeling. Mice received simultaneous intraperitoneal injections of 4‐hydroxytamoxifen (4‐OHT, 50 mg/kg; Sigma–Aldrich, #SML1666) and a low dose of CNO (0.01 mg/kg). Three weeks after tagging, mice were perfused transcardially with PBS followed by 4% paraformaldehyde (PFA). Brains were post‐fixed, cryoprotected in 30% sucrose, and coronally sectioned at 30 µm. 4‐OHT was prepared as previously described [24].

### 5.9 Monosynaptic Anterograde Tracing

A helper of rAAV‐EF1a‐DIO‐EGFP‐T2A‐TK‐WPRE‐hGH polyA, AAV2/8 (2 × 10^1^ ^2^ vg/mL, BrainVTA) was prepared, and 100 nL was injected into the vlPAG/DRN. After 3 weeks of expression, the same site received 150 nL of HSV ΔTK‐hUbC‐tdTomato (BrainVTA). In this system, entry and complementation of the modified HSV depend on TK supplied in starter cells by the helper AAV; the virus propagates anterogradely across a single synapse and labels first‐order postsynaptic neurons with tdTomato. Mice were perfused 5 days after HSV injection, and brains were collected for histological analyses.

### 5.10 Retrograde Tracing

For retrograde labeling, AAVrg‐DIO‐mCherry was injected into the BNST of Vglut2‐Cre or Gad2‐Cre mice. After 3 weeks of expression, mice were perfused, and brains were processed for histological analysis.

### 5.11 Fiber Photometry

For fiber photometry recordings, mice were implanted with an optical fiber (400 µm core, 0.48 NA; Inper) targeted to the vlPAG/DRN. A 3‐week viral expression period was allowed before recording. A fiber photometry system (ThinkerTech) was used to deliver excitation light (470 and 405 nm) and collect GCaMP6 fluorescence signals. Signals were sampled at 100 Hz, and ΔF/F was calculated using standard methods.

### 5.12 Behavioral Testing

Prior to testing, mice were habituated to the experimental room for at least 2 h under dim light (∼20 lux) to minimize anxiety. All experiments and subsequent quantitative analyses were performed by an experimenter blinded to group identity. Some behavior was recorded with a video tracking system (ANY‐maze) and analyzed offline.

#### 5.12.1 Mechanical Sensitivity (von Frey Test)

Mice were placed on an elevated wire mesh grid and acclimated for 20–30 min. The plantar surface of the hind paw was stimulated with a series of calibrated von Frey filaments (0.02–2.56 g) using the up‐down method to determine the paw withdrawal threshold.

#### 5.12.2 Thermal Sensitivity (Hargreaves Test)

Mice were acclimated to the testing apparatus for 1–2 days before the experiment. On the test day, each mouse was placed in a transparent acrylic enclosure on a heated glass floor. A radiant heat source was focused on the plantar surface of the hind paw, and the paw withdrawal latency (PWL) was recorded. A 20 s cutoff was used to prevent tissue damage. Three trials were performed per paw, with at least 5 min between trials, and the average PWL was calculated.

#### 5.12.3 Hot‐Plate Test

Mice were placed on a heated metal plate maintained at 50°C, 53°C, or 56°C. The latency to exhibit nocifensive behaviors (licking, shaking, or jumping of the hind paw) was recorded as the thermal pain threshold. Cutoff times were 60 s (50°C), 30 s (53°C), and 20 s (56°C) to prevent injury. A maximum of three trials per mouse was used for analysis.

#### 5.12.4 Rotarod Test

Mice were trained for 3 consecutive days on a rotating stick set at a speed of 4 rpm until they could remain on the rod for ≥5 min. During testing on an accelerating rotarod (4–40 rpm over 5 min), the latency to fall and the speed at falling were recorded. The best performance of three trials (minimum 10 min inter‐trial interval) was used for analysis.

#### 5.12.5 Real‐Time Place Escape/Avoidance (RT‐PEA)

A two‐chamber apparatus (30 × 28 × 50 cm) with distinct visual cues (black/white walls and stripes) was used. The test consisted of three 10 min phases: (1) Pre‐stimulation: free exploration with no stimulation to determine side preference; (2) Stimulation: optogenetic stimulation (blue or yellow light) or mechanical stimulation (0.4 g von Frey filament) was applied to the hind paw only when the mouse entered its preferred side; (3) Post‐stimulation: free exploration with no stimulation. Avoidance was quantified as the reduction in time spent on the previously preferred side during the Post‐stimulation phase.

#### 5.12.6 Open‐Field Test (OFT)

Mice were placed in the corner of a white acrylic arena (45 × 45 × 45 cm) and allowed to explore freely for 10 min. The arena was cleaned with 70% ethanol between trials. Total distance traveled, number of entries into the center zone (20 × 20 cm), and time spent in the center were analyzed using tracking software (ANY‐maze).

#### 5.12.7 Elevated Plus Maze Test (EPMT)

The maze consisted of two open arms (30 × 6 cm) and two enclosed arms (30 × 6 × 15 cm), connected by a central platform (6 × 6 cm), elevated 1 m above the floor. Mice were placed on the central platform facing an open arm and allowed to explore for 5 min. The maze was cleaned with 70% ethanol between trials. Time spent in and entries into the open arms were analyzed (an entry defined by all four paws entering the arm).

#### 5.12.8 Light‐Dark Box (LDB)

The apparatus consisted of a dark chamber (0 lux) and an illuminated chamber (470–500 lux), separated by a 7 × 7 cm doorway. Mice were placed in the light chamber facing the doorway and allowed to explore for 10 min. Time spent in the light chamber and latency to first enter the dark chamber were recorded.

#### 5.12.9 Sucrose Preference Test (SPT)

Mice were single‑housed and presented with two bottles—one containing 2% sucrose solution and the other plain water. Bottle positions were switched every 24 h during a 48 h acclimation period. After 24 h of water deprivation, mice were given free access to both bottles for 24 h. Sucrose preference was calculated as: [sucrose intake/(sucrose + water intake)] × 100%.

#### 5.12.10 Forced Swim Test (FST)

Mice were placed in a clear plexiglass cylinder (30 cm height, 12 cm diameter) filled with water (23°C–25°C) to a depth of 25 cm for 6 min. The first 2 min were considered acclimation; immobility time (defined as passive floating with only minimal movements to keep the head above water) was scored during the final 4 min.

#### 5.12.11 Tail‐Suspension Test (TST)

Mice were suspended by the tail using adhesive tape, with the head positioned 20–25 cm above the floor, for 6 min. Immobility time (defined as the absence of escape‑directed movements) was scored during the final 4 min.

### 5.13 Quantitative Real‐Time RT‐PCR (qPCR)

Fresh vlPAG/DRN tissues were rapidly collected, snap‐frozen, and total RNA was extracted using the RNeasy Plus Mini Kit (Qiagen). Total RNA (300 ng) was reverse‐transcribed into cDNA using HiScript II Q RT SuperMix for qPCR (+gDNA wiper) (Vazyme). qPCR was performed on a CFX96 Real‐Time RT–PCR system (Bio‐Rad) using ChamQ Universal SYBR qPCR Master Mix (Vazyme). Each 20 µL reaction contained equal amounts of cDNA, 10 µL SYBR Green premix, and 200 nM of each primer. Amplification conditions were: 95°C for 30 s; 40 cycles of 95°C for 5 s and 60°C for 30 s, followed by melt‐curve analysis. Ct values were normalized to *Gapdh*, and relative transcript levels were calculated using the 2^^−ΔΔCt^ method. Primer sequences for mouse *Nlrp3, Il1β, Casp1, Asc*, and *Il18* are listed in Table S2.

### 5.14 Histology and Immunofluorescence

Mice were deeply anesthetized and perfused transcardially with PBS followed by 4% PFA. Brains were post‐fixed in 4% PFA at 4°C for 24 h and cryoprotected in 30% sucrose. Coronal sections (30 µm) were cut on a cryostat.

Free‐floating sections were blocked with 2% BSA and 0.3% Triton X‐100 in PBS for 1–2 h at room temperature. Sections were incubated with primary antibodies overnight at 4°C, washed, and incubated with fluorophore‐conjugated secondary antibodies for 2 h at room temperature. Primary antibodies: goat anti‐Iba1 (1:1000), chicken anti‐GFP (1:1000), guinea pig anti‐c‐Fos (1:1000), and rabbit anti‐RFP (1:1000). Secondary antibodies: species‐specific antibodies conjugated to FITC, Cy3, or Cy5 (1:500; Jackson ImmunoResearch/Abcam). Sections were mounted and imaged using an Olympus FluoView FV1000 confocal microscope or a microscope equipped with a SPOT CCD camera.

### 5.15 Quantitative Analysis of Microglial Morphology and Density

Iba1^+^ or EGFP^+^ microglia in the vlPAG/DRN were imaged by confocal microscopy using a 20× objective under identical acquisition settings. For microglial density analysis, Iba1^+^ or EGFP^+^ cells were counted within a fixed 20× imaging field positioned consistently within the vlPAG/DRN across sections. The vlPAG/DRN region was defined based on the Allen Brain Atlas and anatomical landmarks. Three sections from each mouse were analyzed, and the values from the three sections were averaged to obtain a single value per animal, with the mouse considered the biological replicate.

For morphological analysis, individual microglia with clearly identifiable somata and complete processes within the vlPAG/DRN were selected and analyzed using Fiji/ImageJ. Cells with overlapping processes, truncated processes at the image boundary, or incomplete morphology were excluded. Soma area was measured after image thresholding. For Sholl analysis, the center of the soma was defined as the geometric center of the cell body, and concentric circles were generated from this point with radii increasing at 5 µm intervals. The number of process intersections at each radius and the total number of intersections were quantified using the Sholl analysis plugin as measures of microglial process complexity. Three sections per mouse were analyzed, and cell‐level measurements were first averaged within each animal before statistical analysis. Data are presented as mean ± SEM, with each mouse treated as an independent biological replicate.

### 5.16 Fluorescent In Situ Hybridization (FISH)

#### 5.16.1 Brain Tissues Were Processed Under RNase‐Free Conditions

Coronal sections (18 µm) were prepared and processed using the RNAscope Multiplex Fluorescent Manual Assay Kit (Advanced Cell Diagnostics) according to the manufacturer's protocol. Probes for *Nlrp3, Il1r1, Vglut2*, and *Vgat* are listed in Table S1.

### 5.17 Brain Slice Preparation and Electrophysiology

Mice were anesthetized and transcardially perfused with ice‐cold oxygenated NMDG‐ACSF containing (in mM): 93 NMDG, 2.5 KCl, 0.5 CaCl_2_, 1.2 NaH_2_PO_4_, 30 NaHCO_3_, 5 Na‐ascorbate, 3 Na‐pyruvate, 25 glucose, 20 HEPES, 10 MgSO_4_, 3 glutathione, and 2 thiourea (pH 7.3–7.4). Acute coronal slices (300 µm) containing the vlPAG/DRN were prepared using a vibrating microtome (Leica VT1200 S) at 2°C–4°C.

Slices were incubated in NMDG‐ACSF at 33°C for 10 min, then transferred to HEPES‐ACSF at 25°C for ≥1 h. HEPES‐ACSF contained (in mM): 92 NaCl, 2.5 KCl, 2 CaCl_2_, 1.2 NaH_2_PO_4_, 30 NaHCO_3_, 5 Na‐ascorbate, 3 Na‐pyruvate, 25 glucose, 20 HEPES, 2 MgSO_4_, 3 glutathione, and 2 thiourea. Slices were transferred to a recording chamber continuously perfused with standard ACSF at 32°C containing (in mM): 129 NaCl, 3 KCl, 2.4 CaCl_2_, 20 NaHCO_3_, 10 glucose, 1.3 MgSO_4_, and 1.2 KH_2_PO_4_.

Whole‐cell recordings were obtained using borosilicate glass electrodes (5–8 MΩ) with a MultiClamp 700B amplifier and Digidata 1550B acquisition system. Signals were low‐pass filtered at 2.8 kHz and sampled at 10 kHz. For cytokine treatment experiments, IL‐1β (1 ng/mL) was applied through the perfusion system.

### 5.18 Quantification and Statistical Analysis

Data are presented as mean ± SEM. Statistical analyses were performed using GraphPad Prism 8. Comparisons between two groups were performed using two‐tailed unpaired or paired Student's *t*‐tests, as appropriate. Comparisons among three or more groups were performed using one‐way ANOVA followed by Bonferroni's post hoc test. Two‐way ANOVA followed by Bonferroni's post hoc test was used for comparisons involving two independent variables. A *p*‐value< 0.05 was considered statistically significant (^*^ *p* < 0.05, ^**^ *p* < 0.01, ^***^ *p* < 0.001, ^****^ *p* < 0.0001). ns, not significant.

## Author Contributions

**Jia‐Yi Li**: data curation, investigation, Writing – review and editing, formal analysis, visualization, methodology. **Junping Chen**: resources, writing – review and editing, supervision, project administration. **Shumin Duan**: writing – review and editing, resources, funding acquisition. **Shu‐Lan Xie**: investigation, writing – review and editing, methodology, formal analysis, data curation. **Jing Yang**: investigation, data curation, formal analysis, funding acquisition, writing – original draft, methodology, writing – review and editing, visualization, conceptualization. **Shengmei Zhu**: writing – review and editing, resources, supervision, project administration. **Bing‐Jie Tang**: data curation, investigation, writing – review and editing, formal analysis, methodology. **Zhen‐Zhong Xu**: conceptualization, supervision, project administration, resources, writing – review and editing, methodology, formal analysis, funding acquisition, validation.

## Conflicts of Interest

The authors declare no conflicts of interest.

## Supporting information




**Supporting File**: advs77071‐sup‐0001‐SuppMat.pdf.

## Data Availability

Data that support the findings of this study are available from the corresponding author upon reasonable request.
