# Supplementary material for: VlPAG/DRN Microglia Drive Neuropathic Pain‐Induced Depression via a Defined Neuroimmune Axis
Source: Adv Sci (Weinh). 2026 Aug 11;13(61):e77071. doi: 10.1002/advs.77071 (PMC13460408; doi:10.1002/advs.77071)
Supplement: Supplementary file 1 [file ADVS-13-e77071-s001.pdf]

Supplemental Materials for

vlPAG/DRN microglia drive neuropathic pain-induced depression via a defined neuroimmune axis

Jing Yang[1,2,3,4,7*], Bing-Jie Tang[1,5,6,7], Jia-Yi Li[3,7], Shu-Lan Xie[1,5,6,7], Shumin Duan[1,5,6], Junping Chen[3,4*], Shengmei Zhu[1*], Zhen-Zhong Xu[1,2,5,6*]

Figure S1- Figure S8

Table S1-S2

SUPPLEMENTARY FIGURES and FIGURE LEGENDS

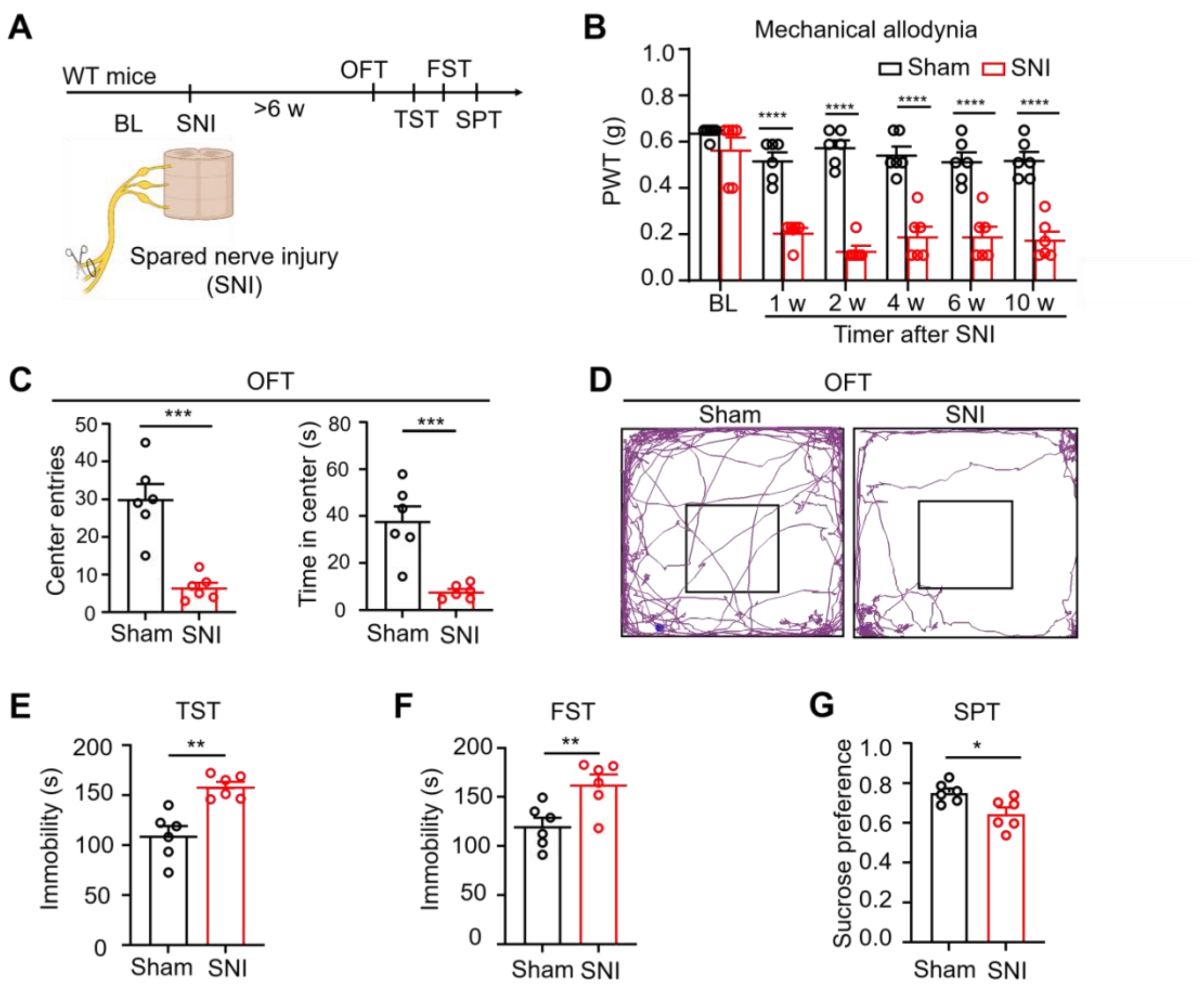

Figure S1. SNI induces robust neuropathic pain, anxiety, and depression-like behaviors.

(A) Experimental timeline for spared nerve injury (SNI) and behavioral testing in WT mice.

(B) Paw withdrawal thresholds (PWT) show the development of mechanical allodynia in sham and SNI mice over time after surgery (sham, n = 6; SNI, n = 6).

(C and D) Anxiety-like behaviors assessed by the open-field test (OFT) (C), with representative trajectories shown in (D) (sham, n = 6; SNI, n = 6).

(E-G) Depression-like behaviors assessed by the tail-suspension test (TST) (E), forced swim test (FST) (F) and sucrose preference test (SPT) (G) in sham and SNI mice (sham, n = 6; SNI, n = 6).

ns, not significant. *P < 0.05, **P < 0.01, ***P < 0.001, ****P < 0.0001. Data in (B) were analyzed by two-way ANOVA with Bonferroni's post hoc analysis; data in (C–G) were analyzed by unpaired t test. Data are presented as mean ± SEM.

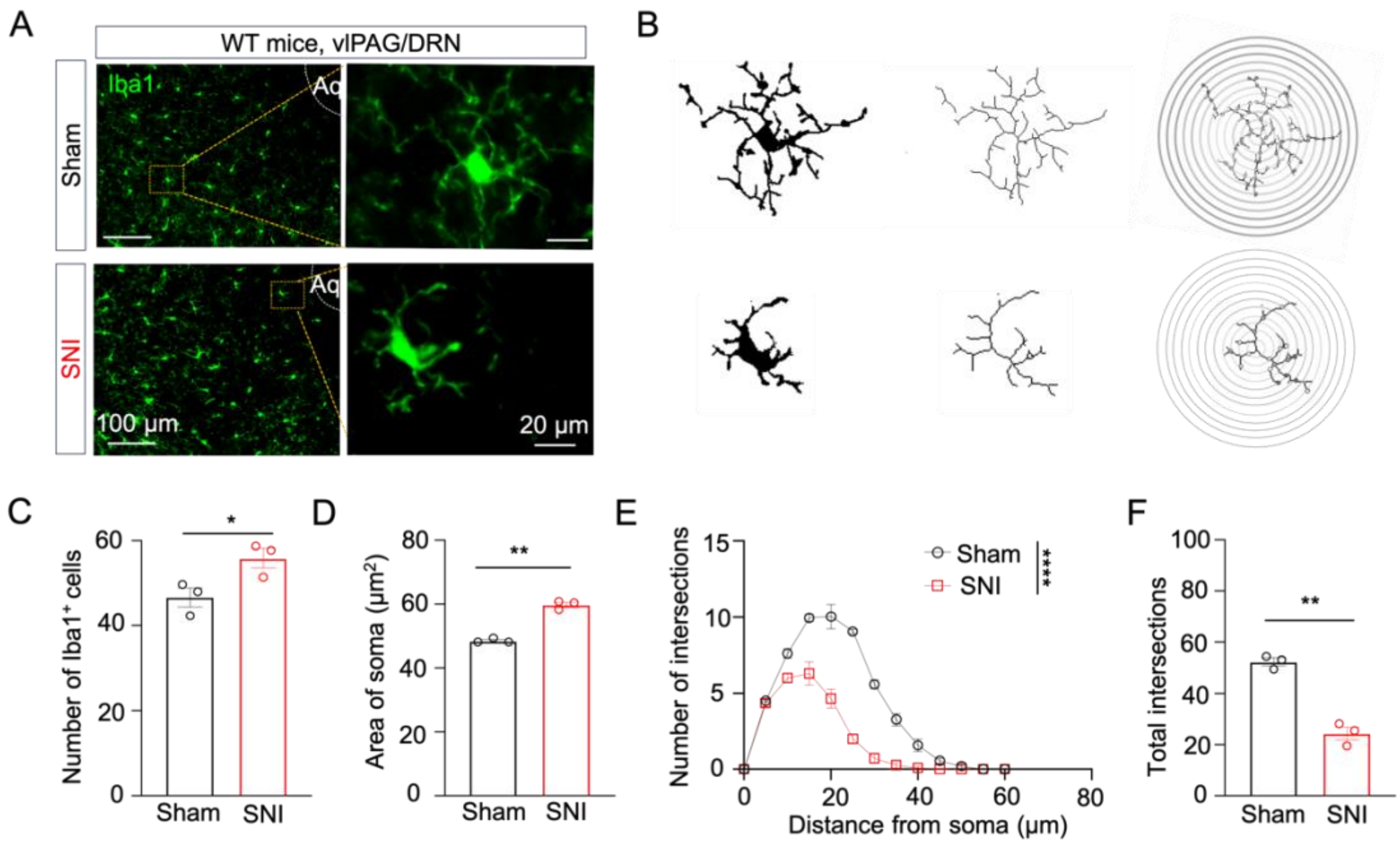

Figure S2. Quantitative analysis of microglial morphology and density in the vlPAG/DRN after SNI.

(A) Representative immunofluorescence images of $Iba1^{+}$ microglia in the vlPAG/DRN under sham and SNI conditions. Scale bars, 100 μm (left) and 20 μm (right).

(B) Representative single microglia images before and after image processing for Sholl analysis.

(C) Quantification of microglial cell density in sham and SNI mice.

(D) Quantification of somatic area (μm²) of individual microglia in sham and SNI mice.

(E) Sholl analysis showing the number of intersections as a function of distance from the soma in sham and SNI mice.

(F) Quantification of total intersections (cumulative Sholl intersections) comparing sham and SNI groups.

Data are presented as mean ± SEM. For each group, $n = 3$ mice, with each mouse-level data point representing the average of 3 sections per mouse. *$P < 0.05$, **$P < 0.01$, **** $P < 0.0001$; unpaired t-test for (C, D, F), two-way ANOVA with Bonferroni post hoc test for (E).

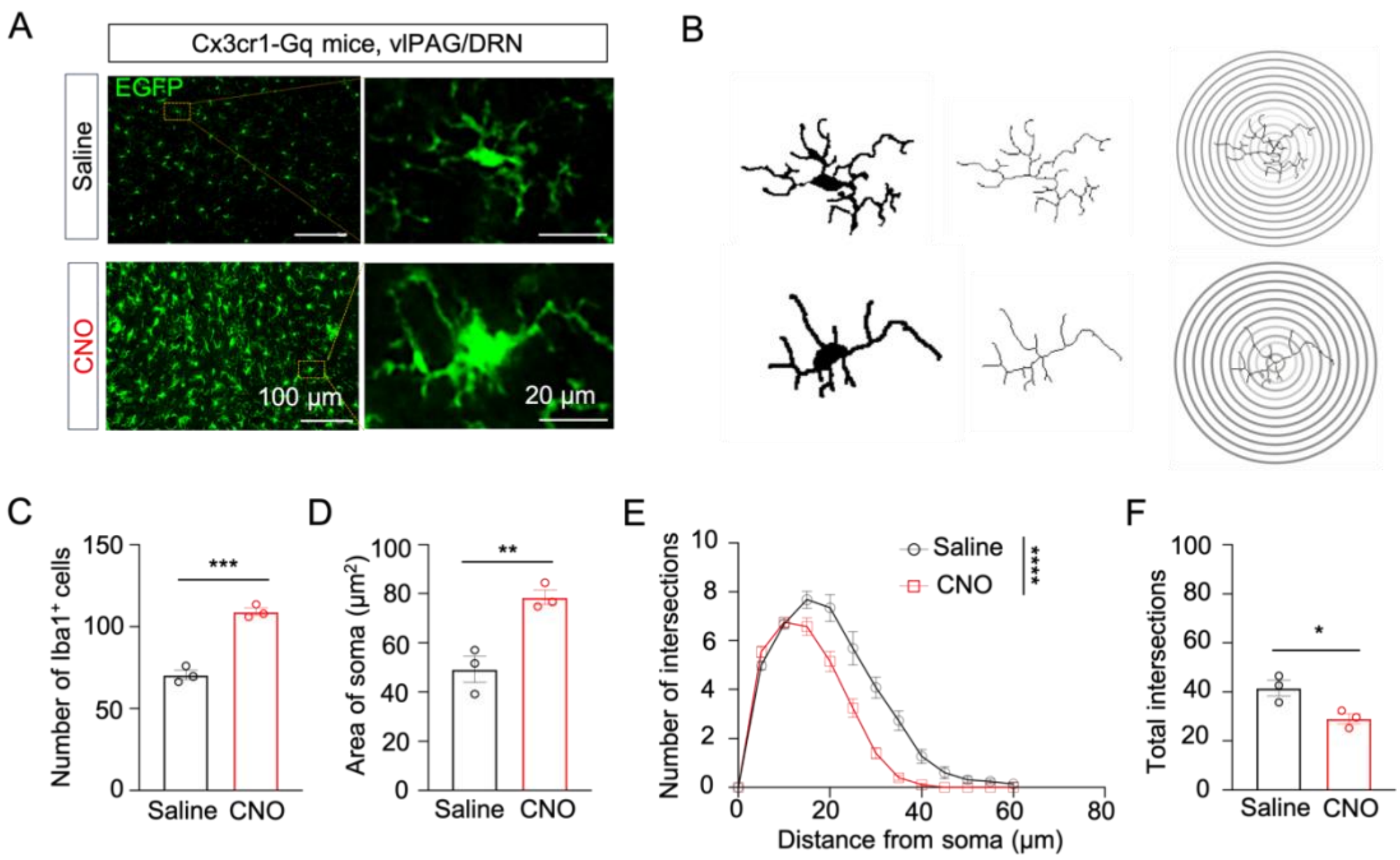

Figure S3. Quantitative analysis of microglial morphology and density in the vlPAG/DRN after chemogenetic activation in Cx3cr1-Gq mice.

(A) Representative immunofluorescence images of $Iba1^+$ microglia in the PAG under saline and CNO conditions. Scale bars, 100 μm (left) and 20 μm (right).

(B) Representative single microglia images before and after image processing for Sholl analysis.

(C) Quantification of microglial cell density in saline and CNO treated mice.

(D) Quantification of somatic area (μm²) of individual microglia in saline and CNO treated mice.

(E) Sholl analysis showing the number of intersections as a function of distance from the soma in saline and CNO treated mice.

(F) Quantification of total intersections (cumulative Sholl intersections) comparing saline and CNO treated groups.

Data are presented as mean ± SEM. For each group, n = 3 mice, with each mouse-level data point representing the average of 3 sections per mouse. *P < 0.05, **P < 0.01, ***P < 0.001, **** P < 0.0001; unpaired t-test for (C, D, F), two-way ANOVA with Bonferroni post hoc test for (E).

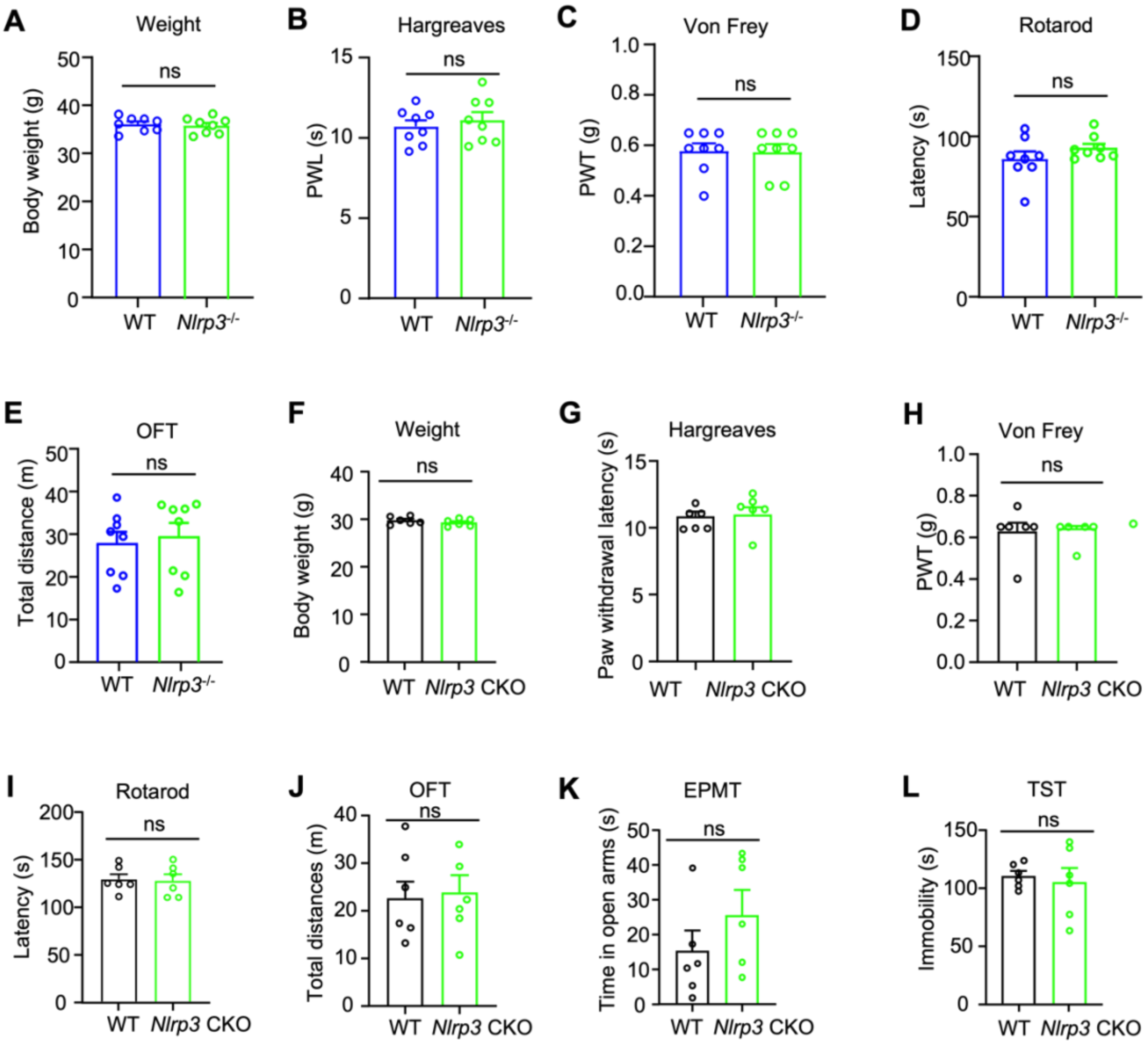

Figure S4. Baseline behaviors and motor functions are normal in *Nlrp3* knockout mice.

(A–E) Baseline assessments in WT and *Nlrp3*$^{-/-}$ mice, including body weight (A), thermal sensitivity (Hargreaves test, B), mechanical threshold (von Frey test, C), motor coordination (rotarod test, D), and locomotor activity (open-field test, E) (WT, n = 8; *Nlrp3*$^{-/-}$, n = 8).

(F–L) Baseline assessments in WT and microglia-specific *Nlrp3* conditional knockout (*Nlrp3* CKO) mice, including body weight (F), thermal sensitivity (G), mechanical threshold (H), motor coordination (I), locomotor activity (J), anxiety-like behavior (elevated plus maze test, K), and depression-like behavior (tail-suspension test, L) (WT, n = 6; *Nlrp3* CKO, n = 6).

ns, not significant. Data were analyzed by unpaired t test and are presented as mean ± SEM.

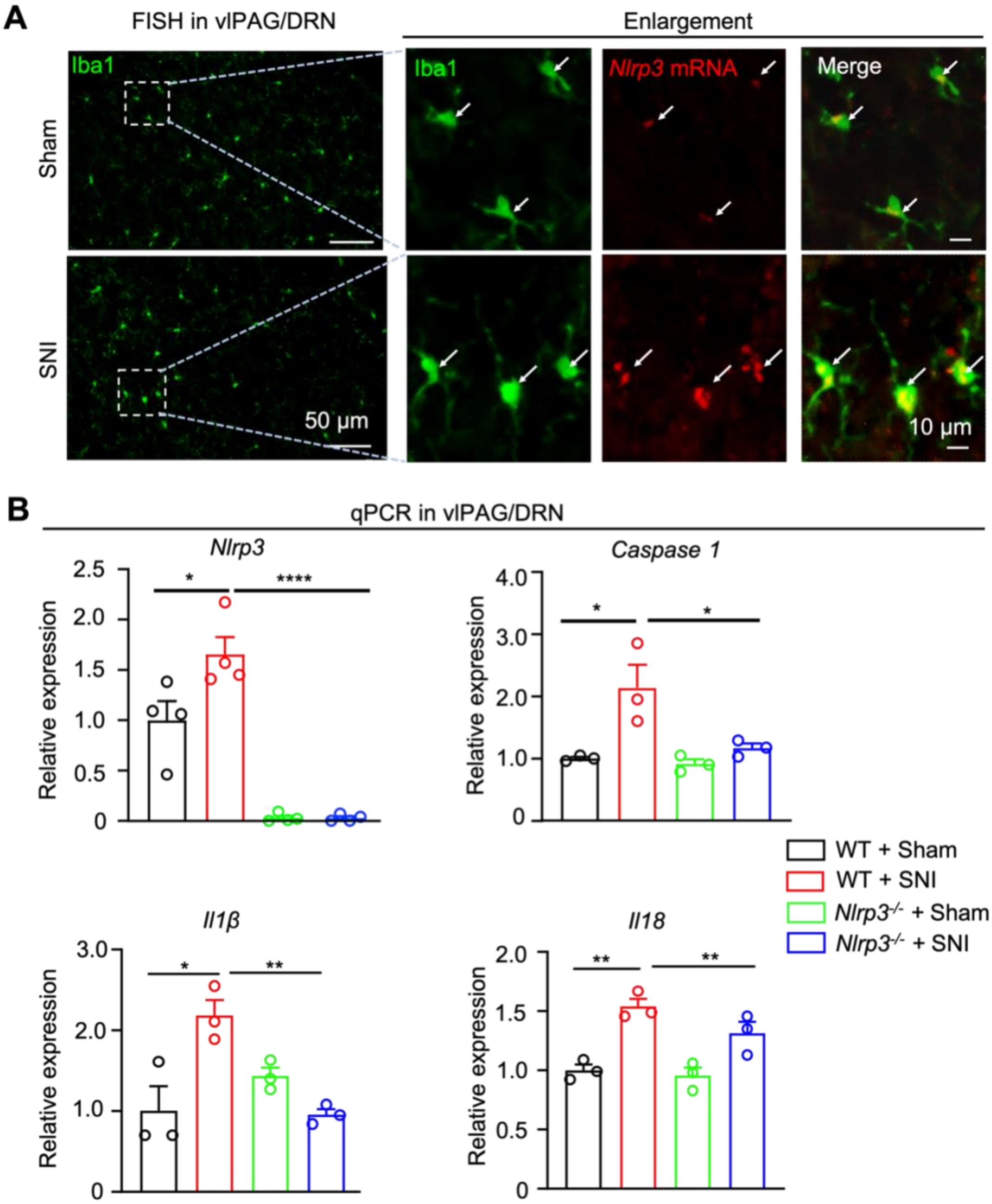

Figure S5. Microglial NLRP3 in the midbrain is critical for neuropathic pain-induced inflammatory signaling.

(A) Representative fluorescent *in situ* hybridization (FISH) images showing Iba1^{+} microglia (green) and *Nlrp3* mRNA (red) in the vlPAG/DRN of WT mice after sham or SNI surgery, with enlarged views. Arrows indicate *Nlrp3* mRNA in Iba1^{+} microglia . Scale bars, 50 μm (left) and 10 μm (right).

(B) qPCR analysis of *Nlrp3*, *Caspase 1*, *Il1β*, and *Il18* mRNA levels in the vlPAG/DRN of WT and *Nlrp3*$^{-/-}$ mice with sham or SNI surgery (n = 3-4 mice/group).

ns, not significant. *P < 0.05, **P < 0.01, ****P < 0.0001. Data were analyzed by one-way ANOVA with Bonferroni's post hoc test and are presented as mean ± SEM.

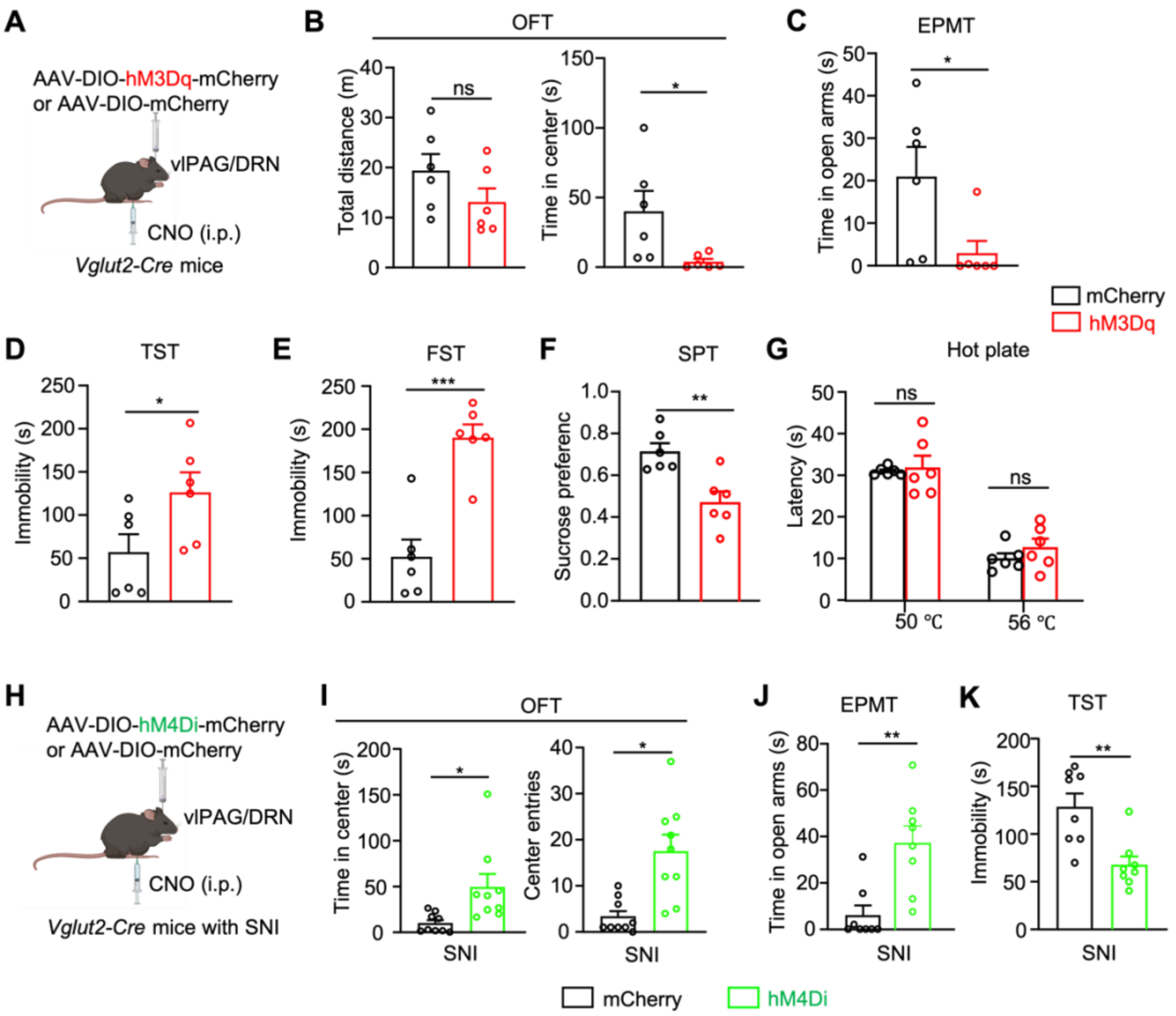

Figure S6. Chemogenetic manipulation of vlPAG/DRN VGLUT2⁺ neurons bidirectionally modulates affective behaviors.

(A) Experimental strategy for chemogenetic activation of VGLUT2⁺ neurons in the vlPAG/DRN.

(B and C) Open-field test (OFT) (B) and elevated plus maze test (EPMT) (C) following chemogenetic activation of vlPAG/DRN VGLUT2⁺ neurons (mCherry, n = 6; hM3Dq, n = 6).

(D–F) Affective behaviors assessed by the tail-suspension test (TST) (D), forced swim test (FST) (E), and sucrose preference test (SPT) (F) following CNO administration (mCherry, n = 6; hM3Dq, n = 6).

(G) Hot-plate test to assess nociceptive responses following vlPAG/DRN neuronal activation (mCherry, n = 6; hM3Dq, n = 6).

(H) Experimental strategy for chemogenetic inhibition of VGLUT2⁺ neurons in the vlPAG/DRN.

(I–K) Open-field test (OFT) (I), elevated plus maze test (EPMT) (J), and tail-suspension test (TST) (K) following chemogenetic inhibition of vlPAG/DRN VGLUT2⁺ neurons

in SNI mice (mCherry, n = 8-9; hM4Di, n = 8-9).

ns, not significant. *P< 0.05, **P < 0.01,***P < 0.001. Data were analyzed by unpaired t test and are presented as mean ± SEM.

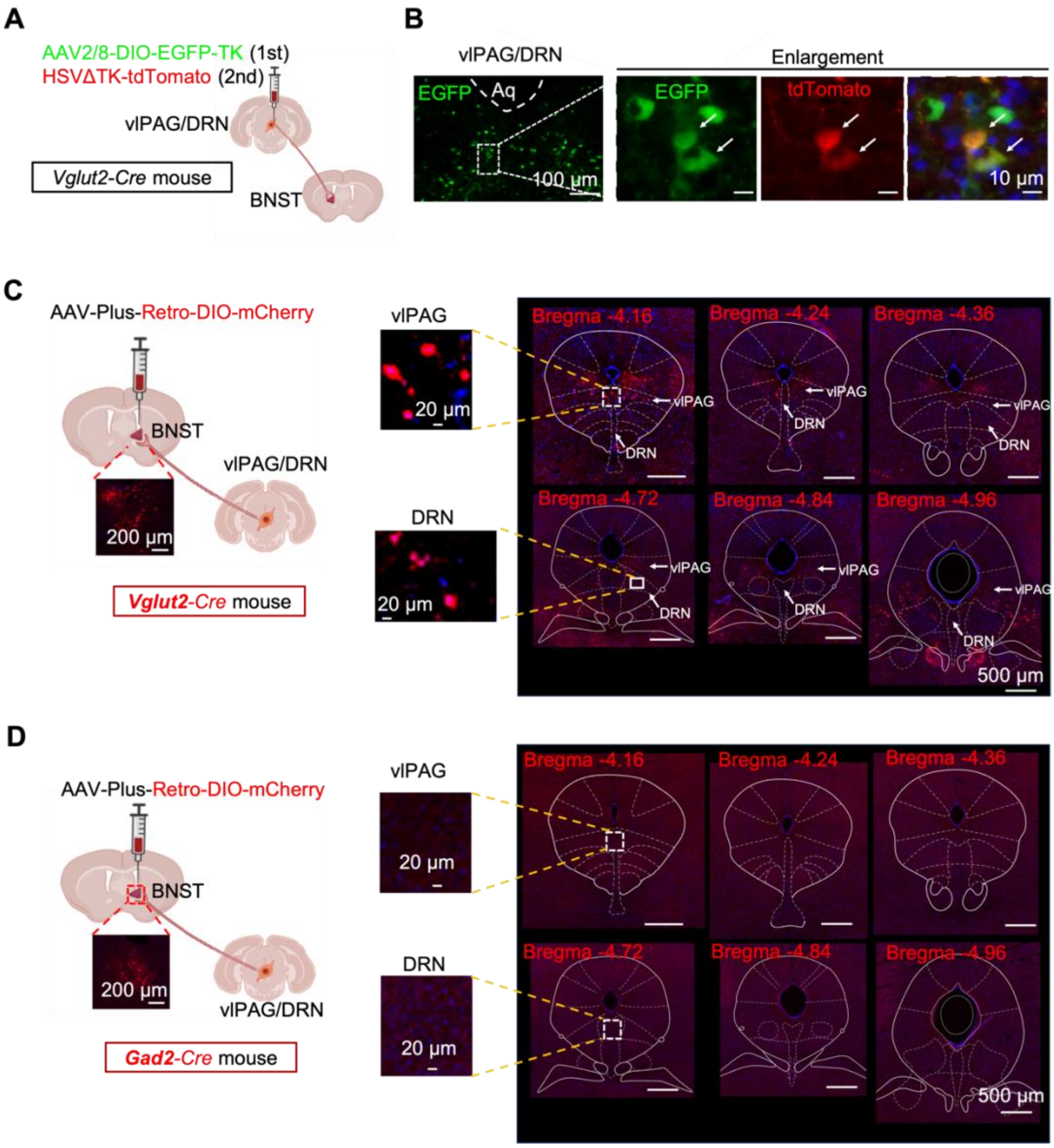

Figure S7. Circuit mapping of vlPAG/DRN^{Glu} projections to BNST neurons.

(A) Viral strategy for HSV-based monosynaptic anterograde tracing from vlPAG/DRN^{Glu} neurons to BNST neurons in *Vglut2*-Cre mice.

(B) Representative images in the vlPAG/DRN showing EGFP and tdTomato signals with enlarged views; arrows indicate double-labeled starter neurons. Scale bars, 100 μm (left) and 10 μm (right).

(C) Retrograde labeling strategy (AAV-Plus-Retro-DIO-mCherry injected into BNST) and representative images showing abundant retrogradely labeled mCherry^{+} somata in the vlPAG/DRN (Bregma −4.16 to −4.96 mm) in Vglut2-Cre mouse. Scale bars, 20 μm (insets) and 500 μm (overview).

(D) Same retrograde labeling strategy in Gad2-Cre mouse showing sparse mCherry^{+} somata in the vlPAG/DRN at corresponding levels. Scale bars, 20 μm (insets) and 500 μm (overview).

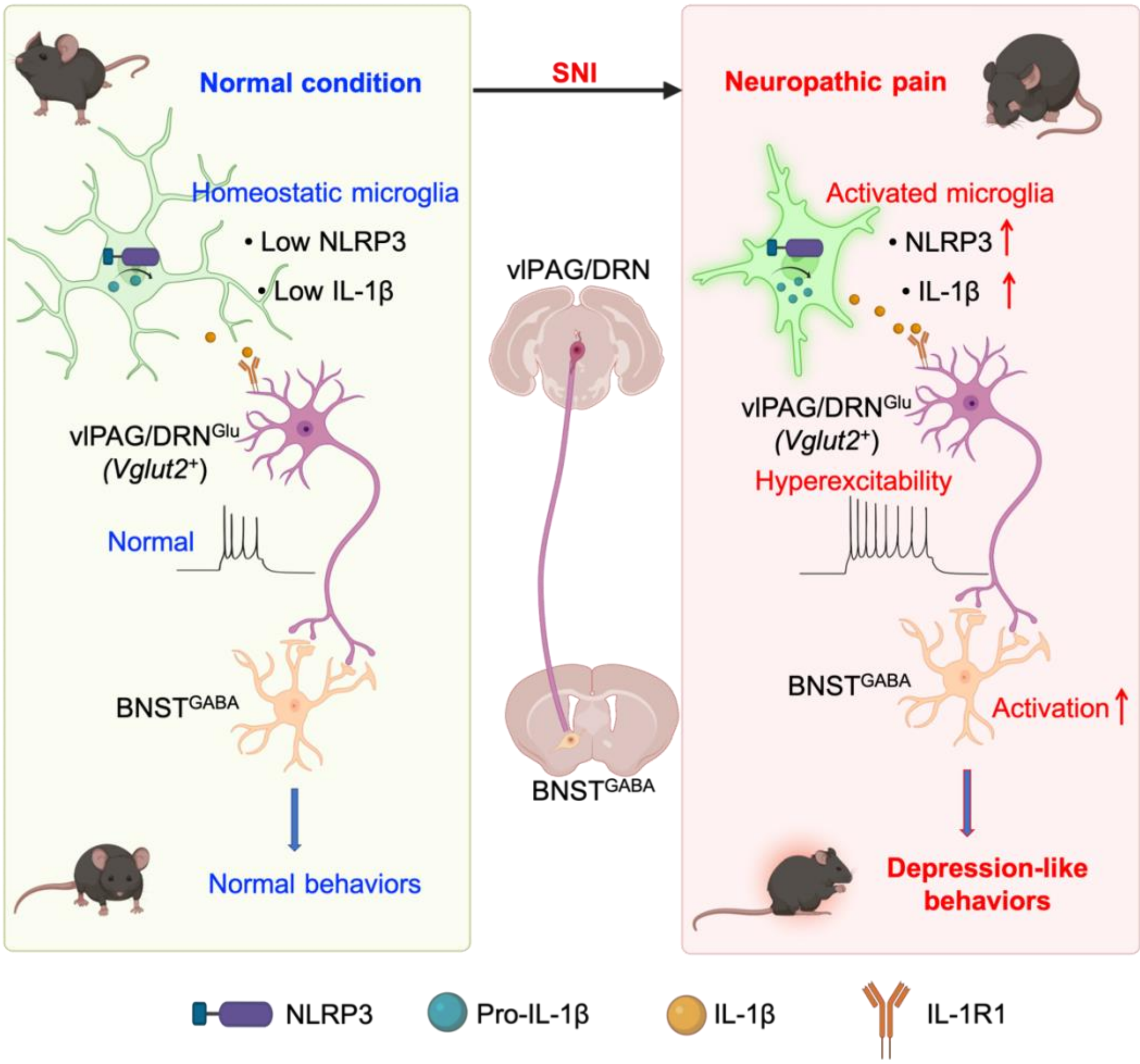

Figure S8. Proposed working model for microglia in the vlPAG/DRN drive neuropathic pain-induced depression via a neuroimmune axis.

Peripheral nerve injury (SNI) induces neuropathic pain, activates midbrain microglia, and triggers an NLRP3–IL-1β–dependent inflammatory cascade. IL-1β signals through IL-1R1 on vlPAG/DRN VGLUT2^{+} neurons (vlPAG/DRN^{Glu}), inducing hyperexcitability and driving the vlPAG/DRN^{Glu}→BNST^{GABA} circuit to produce the affective component of neuropathic pain, including depression-like behaviors.

Table S1. The key resources and reagents used in the present study.

Reagent or resource	Source	Identifier
**Reagents**		
Recombinant IL-1 beta Protein	R&D Systems	Cat# 201-LB-100/CF
Recombinant IL-1Ra Protein	R&D Systems	Cat# 280-RA-050/CF
Tamoxifen	Sigma-Aldrich	Cat# T5648
4-hydroxytamoxifen	Sigma-Aldrich	Cat# SML1666
DMEM/F12	Gibco	Cat# 11320082
HBSS	Gibco	Cat# 14025092
B27	Gibco	Cat# 17504044
Clozapine N-oxide dihydrochloride	MCE	Cat# HY-17366A
**Viral vectors**		
rAAV-hSyn-DIO-GCaMp6s-WPREs	BrainVTA	Cat# PT-0091
AAV-hSyn-FLEx-mGFP-2A-Synaptophysin-mRuby	Taitool	Cat# S0250-9
AAV2/9-mCaMKIIa-hM3D(Gq)-EGFP-WPRE-pA	Taitool	Cat# S1217-9
AAV2/9-hSyn-DIO-hM3D(Gq)-mCherry-WPRE-pA	Taitool	Cat# S0192-9
AAV2/9-hSyn-DIO-mCherry-pA	Taitool	Cat# S0240-9
AAV2/1-mCaMKIIa-Cre-WPRE-pA	Taitool	Cat# S0906-1
AAV2/9-hSyn-DIO-hM3D(Gq)-ER2-P2A-EGFP-WPRE-pA	Taitool	Cat# S1272-9
AAV2/9-hSyn-DIO-EGFP-WPRE-pA	Taitool	Cat# S0746-9
AAV2/9-hSyn-DIO-hChR2(H134R)-mCherry-ER2-WPRE-PA	Taitool	Cat# S0479-9
AAV2/9-hSyn-DIO-mCherry-pA	Taitool	Cat# S0240-9
AAV-retro-plus-hSyn-DIO-mcherry-pA	Taitool	Cat# S0240-2RP
AAV2/9-hEF1a-DIO-NpHR-EYFP	Vector Core at ZJU	N/A
AAV2/9-hEF1a-DIO-EYFP	Vector Core at ZJU	N/A
rAAV-EF1a-DIO-EGFP-T2A-TK-WPRE-hGH polyA, AAV2/8	BrainVTA	Cat# PT-0087
HSV ΔTK-hUbC-tdTomato	BrainVTA	Cat# H03001
pAAV-hSyn-Cre-WPRE	Obio Technology	Cat# CN867
pAAV-GAD67-DIO-hM3D(Gq)-mCherry-CW3SL	Obio Technology	Cat# H15038
AAV2/9-shRNA-scramble	Shanghai Sunbio Medical Biotechnology	pAAVE3649
AAV2/9-shRNA-*Il1r1*	Shanghai Sunbio Medical Biotechnology	pAAVE3650
**Antibodies**		
Goat anti-Iba1	Novus Biologicals	Cat# NB100-1028

Guinea pig anti-c-Fos	Oasis Biofarm	Cat# OB-PGP080
Chicken anti-EGFP	Oasis Biofarm	Cat# OB-PRB014
Rabbit anti-RFP	ROCKLAND	Cat# 600-401-379
DAPI	Vector laboratories	Cat# H-1200
Donkey anti-rabbit IgG H&L-Cy3	Jackson	Cat# 711-165-152
Donkey anti-goat IgG H&L-488	Abcam	Cat# ab150129
Donkey anti- Guinea pig IgG H&L-Cy5	Abcam	Cat# ab150187
Donkey anti-chicken IgG H&L-488	Jackson	Cat# 703-545-155
***In situ* Hybridization Probes**		
RNAscope multiplex fluorescent manual assay kit	ACDBio	Cat# 323100
RNAscope Probe-Mm-*Nlrp3*	ACDBio	Cat# 316791
RNAscope Probe-Mm-*Vglut2*	ACDBio	Cat# 405011
RNAscope Probe-Mm-*Illr1*	ACDBio	Cat# 415851
**Mouse strains**		
Nlrp3$^{-/-}$ mouse	Jackson laboratory	Stock #: 021302
Nlrp3$^{fl/fl}$ mouse	This study	N/A
Cx3cr1^{CreER} mouse	Jackson laboratory	Stock #: 021160
Ai14 (RCL-tdT)-D mouse	Jackson laboratory	Stock #: 007914
Ai32 (RCL-ChR2(H134R)/EYFP) mouse	Jackson laboratory	Stock #:012569
Ai96 (Rosa^{GCamp6s}) mouse	Jackson laboratory	Stock #:028866
Vglut2-Cre mouse	Jackson laboratory	Stock#: 016963
Fos$^{2A-iCreER}$ (TRAP2) mouse	Jackson laboratory	Stock #: 030323
Gq (R26-LSL-hM3Dq-DREADD) mouse	Jackson laboratory	Stock #: 030323

Table S2. The oligonucleotides used for qPCR in the present study.

Genes	Primer sequences
Gapdh	F: 5'-AGGTCGGTGTGAACGGATTTG-3'<br>R: 5'-GGGGTCGTTGATGGCAACA-3'
Nlrp3	F: 5'-GCTCCAACCATTCTCTGACCA-3'<br>R: 5'-GGTTGGTTTTGAGCACAGAGG-3'
Caspase1	F: 5'-GAGCTGATGTTGACCTCAGAG-3'<br>R: 5'- CTGTCAGAAGTCTTGTGCTCTG-3'
Iba1	F: 5'-GCTTTTGGACTGCTGAAGGC-3'<br>R: 5'-GTTTGGACGGCAGATCCTCA-3'
Il1β	F: 5'- GCCTGTGTTTTCCTCCTTGC-3'<br>R: 5'-TGCTGCCTAATGTCCCCTTG-3'
Il18	F: 5'-CAAAGTTAGGTGGGGAGGGTT-3'<br>R: 5'-TGGAAATACAGGCGAGGTCA-3'
Il1r1	F: 5'-ATACCAGATGCCAGCCCAAC-3'<br>R: 5'-GCACAGGTTTGGTTCGTGAC-3'
